# Extracellular Vesicles in Osteogenesis: A Comprehensive Review of Mechanisms and Therapeutic Potential for Bone Regeneration

**DOI:** 10.3390/cimb47080675

**Published:** 2025-08-21

**Authors:** Sreyee Biswas, Prakash Gangadaran, Chandrajeet Dhara, Shreya Ghosh, Soumya Deep Phadikar, Akash Chakraborty, Atharva Anand Mahajan, Ranit Mondal, Debdeep Chattopadhyay, Trisha Banerjee, Anuvab Dey, Subhrojyoti Ghosh, Anand Krishnan, Byeong-Cheol Ahn, Ramya Lakshmi Rajendran

**Affiliations:** 1 Department of Biotechnology, Heritage Institute of Technology, Kolkata 700107, West Bengal, India; sreyeebiswas@gmail.com; 2 Department of Nuclear Medicine, School of Medicine, Kyungpook National University, Daegu 41944, Republic of Korea; prakashg@knu.ac.kr; 3 Cardiovascular Research Institute, Kyungpook National University, Daegu 41944, Republic of Korea; 4 School of Biosciences, Apeejay Stya University, Sohna-Palwal Road, Sohna, Gurugram 122103, Haryana, India; chandudhara2804@gmail.com; 5 Department of Microbiology, St. Xavier’s College, Kolkata 700016, West Bengal, India; shreyaghosh11813@gmail.com (S.G.); trishabanerjee947@gmail.com (T.B.); 6 Department of Chemistry and Chemical Biology, Indian Institute of Technology (ISM), Dhanbad 826004, Jharkhand, India; soumyadeepphadikar3@gmail.com; 7 Department of Biotechnology, Indian Institute of Technology Madras, Chennai 600036, Tamil Nadu, India; akashchakraborty138@gmail.com (A.C.); subhrojyotighosh8@gmail.com (S.G.); 8 Advanced Centre for Treatment, Research and Education in Cancer, Navi Mumbai 410210, Maharashtra, India; atharva66mahajan@gmail.com; 9 Department of Pharmaceutical Technology, Jadavpur University, Kolkata 700032, West Bengal, India; ranitmondal30@gmail.com; 10 Department of Biotechnology, St. Xavier’s College, Kolkata 700016, West Bengal, India; chattopadhyay.debdeep777@gmail.com; 11 Department of Biosciences and Bioengineering, Indian Institute of Technology Guwahati, North Guwahati 781039, Assam, India; anuvab2000dey@gmail.com; 12 Precision Medicine and Integrated Nano-Diagnostics (P-MIND) Research Group, Office of the Dean, Faculty of Health Sciences, University of the Free State, Bloemfontein 9300, South Africa; krishnana1@ufs.ac.za; 13 BK21 FOUR KNU Convergence Educational Program of Biomedical Sciences for Creative Future Talents, Department of Biomedical Sciences, School of Medicine, Kyungpook National University, Daegu 41944, Republic of Korea; 14 Department of Nuclear Medicine, Kyungpook National University Hospital, Daegu 41944, Republic of Korea

**Keywords:** extracellular vesicles, osteogenesis, bone regeneration, mesenchymal stem cells, fracture healing, osteoporosis, tissue engineering, angiogenesis, immunomodulation, biomaterials

## Abstract

Extracellular vesicles (EVs) are nanoscale, membrane-bound particles secreted by diverse cell types and act as pivotal mediators of intercellular communication during bone regeneration. These vesicles transport bioactive cargo including proteins, lipids, mRNAs, and microRNAs that modulate osteogenesis, angiogenesis, and immune responses within the bone microenvironment. EVs originating from mesenchymal stem cells, osteoblasts, endothelial cells, and macrophages have demonstrated substantial potential to promote bone formation, inhibit bone resorption, and enhance vascularization. This review examines the biogenesis, classification, and cellular uptake mechanisms of EVs, focusing on their roles in osteogenesis and their therapeutic applications in fracture healing, osteoporosis, and bone tissue engineering. Despite their promise, significant challenges remain, including the need for standardization, scalable production, and assessment of long-term safety to enable clinical translation of EV-based therapies. Here, we provide a comprehensive overview of EV biology, elucidate the molecular mechanisms of EVs in bone regeneration, and discuss innovative strategies to optimize their therapeutic efficacy, highlighting their potential as next-generation orthobiologics.

## 1. Introduction

Bone regeneration is a complex and tightly regulated process that requires coordination among osteoblasts, osteoclasts, and various signaling molecules. Disorders such as osteoporosis, fractures, and critical bone defects significantly impair natural bone healing. While autografts are limited in availability and synthetic substitutes often exhibit poor bioactivity, small extracellular vesicles (sEVs) facilitate tissue regeneration by delivering bioactive signals. The concept of “morphology memory” in sEVs suggests that these vesicles retain cues from nanostructured biomaterials. Notably, sEVs derived from mesenchymal stem cells (MSCs) cultured on nanostructured titanium (Ti8-21-sEV) have been shown to enhance osteogenesis both in vitro and in vivo, representing a promising cell-free approach for bone regeneration [1,2].

Orthobiologics, including various growth factors such as bone morphogenetic proteins (BMPs), have been employed to enhance bone regeneration and accelerate healing. In the treatment of bone fractures, these agents can reduce treatment duration. Clinically, they are applied either alone or in combination with bone grafts. However, concerns remain regarding risks such as ectopic bone formation and the high cost of treatment [3].

Young osteocyte-derived EVs (YO-EVs) stimulate osteogenesis in bone marrow stromal cells (BMSCs) by increasing alkaline phosphatase activity, mineralization, and expression of osteogenic genes. In contrast, senescent osteocyte-derived EVs (SO-EVs) induce adipogenic differentiation in BMSCs. Neither YO-EVs nor SO-EVs affect osteoclastogenesis. In transgenic mice, osteocyte-derived EVs were detected within the bone matrix, and mature osteoclasts released these vesicles from bone slices. Intravenous administration of YO-EVs increased bone mass and strength, while SO-EVs promoted bone marrow adiposity. Proteomic analyses revealed that tropomyosin-1 (TPM1) is enriched in YO-EVs, increasing BMSC matrix stiffness and osteogenesis; depletion of Tpm1 abrogates these effects [4].

MSC-derived EVs regulate osteoblast activity, and reciprocally, osteoblast-derived EVs influence MSCs, establishing a feedback loop that supports bone healing. For instance, EVs mediate communication between osteoblasts and osteoclasts via RANKL-RANK signaling. Osteoblasts activated by mechanical loading release osteogenic EVs. Additionally, MSC-derived EVs modulate immune responses by promoting anti-inflammatory cell phenotypes through specific miRNAs, whereas immune cell-derived EVs can influence MSC recruitment and differentiation. Collectively, EVs are essential regulators of bone regeneration due to their central role in intercellular communication [5].

## 2. Biology of EVs

### 2.1. Classification of EVs

EVs are classified according to several parameters: classification by cellular origin. Exosomes originate within the cell, forming from endosomes, and are released when these compartments fuse with the plasma membrane. They function as intracellular packages that are subsequently secreted [6,7]. Exosomes can contain DNA and histone proteins, typically confined to the nucleus. Their release may occur actively—via autophagy pathways involving LC3+ amphisomes—or passively during cell death [6]. Exosome production involves biogenesis, transport, and release [8]. Recent studies have identified two size-based exosome subtypes in immune-like cells derived from the human THP-1 cell line: (i) Exo-S: small (40–80 nm) and CD63-rich, and (ii) Exo-L: larger (80–150 nm) and CD9-enriched. Microvesicles form directly at the plasma membrane, where a portion of the membrane buds outward and pinches off, producing small membrane-bound vesicles [9]. During programmed cell death (apoptosis), cells fragment into apoptotic bodies [6]. Autophagic EVs arise during autophagy, where cellular components are repackaged into vesicles for export [6].

### 2.2. Classification by Functional Roles

Oncosomes: they are secreted by cancer cells, and these EVs are typically large and contain tumor-supportive components [10]. Matrix vesicles: they are associated with mineralization processes, particularly in bone tissue [11]. Stress EVs: they are released in response to cellular stress [12]. Migrasomes: they are generated during cell migration [13]. Not all secreted particles are EVs. Exomeres are nonvesicular nanoparticles lacking a membrane. They transport molecules such as ST6Gal-I and amphiregulin (AREG), which can modulate neighboring cells [14]. For example, ST6Gal-I delivered to recipient cells can induce a cancer stem cell-like phenotype [15].

### 2.3. EV Size Categories

Small EVs (sEVs): Typically, they are less than 200 nm, and they primarily include exosomes. A distinct subclass of ARMMs (ARRDC1-mediated microvesicles, ~40–100 nm) is secreted via a pathway involving ARRDC1 and TSG101. Large EVs: they are greater than 200 nm, and this group encompasses large microvesicles and oncosomes [16,17,18]. Large EVs are further subdivided into the following categories: (i) classic large microvesicles (150–1000 nm) and (ii) Annexin A1-positive large oncosomes (1–10 μm)

### 2.4. Biogenesis and Composition of EVs

The multi-step process of small EV (sEV) biogenesis and release is tightly regulated by numerous cellular signals and proteins, particularly during exosome formation [19]. Biogenesis initiates at plasma membrane regions rich in phosphatidylserine [20], where membrane invagination leads to early endosome formation via a calcium-dependent endocytic mechanism [21]. Annexins facilitate the formation of early endosomes [22]. Subsequently, early endosomes mature into late endosomes, within which intraluminal vesicles (ILVs) bud inward. These ILVs accumulate within multivesicular bodies (MVBs), typically 30–100 nm in diameter [23]. Flotillin is incorporated during vesicle invagination, while SNARE complexes mediate fusion of MVBs with target membranes [24]. MVB formation is orchestrated by the endosomal sorting complex required for transport (ESCRT), a multi-protein machinery comprising four subunits: (i) ESCRT-0 mediates recruitment of ubiquitinated cargo, (ii) ESCRT-I and ESCRT-II facilitate membrane budding, and (iii) ESCRT-III enables vesicle scission and release [25].

Other key components include VPS4, VTA1, ALIX [26], Tsg101, tetraspanins (CD63, CD9, CD81, CD82), RAB GTPases, heat shock proteins (HSP-70, HSP-90), and cell adhesion molecules [27]. After maturation, MVBs follow one of the following two fates: fusion with lysosomes for degradation or fusion with the plasma membrane to release ILVs (now termed exosomes) extracellularly [26]. Release is regulated by RAB proteins and GTPases. Notably, cells can generate MVBs via ESCRT-independent mechanisms, though these alternative pathways remain under investigation [28]. Exosome secretion is also modulated by environmental factors such as pH shifts, hypoxia, oxidative stress, heat shock, and irradiation [29]. The stress-activated protein p53 has been shown to increase exosome release [30]. In summary, exosome biogenesis is a sophisticated, highly regulated process involving numerous molecular participants and signaling pathways. The cargo and properties of exosomes reflect the microenvironment and cellular lineage of their origin, underscoring their value as intercellular messengers in both physiological and pathological contexts. Please refer to Table 1 for detailed understanding.

### 2.5. Mechanisms of EV Uptake and Cell Interaction

EVs function as dynamic mediators of intercellular communication by transferring biologically active molecules—including proteins, mRNA, and miRNA—between cells. Both direct and indirect evidence support that EVs are internalized by recipient cells and deliver functionally active cargo. For instance, EVs have successfully transferred mRNA and miRNA from mouse cells to human mast cells, resulting in detectable mouse protein production in the recipient human cells [38]. Likewise, EVs engineered to carry siRNA have been shown to silence specific genes in recipient cells [39]. Another study reported that EVs loaded with the substrate luciferin induced bioluminescence in luciferase-expressing cells, providing the first evidence of efficient cytoplasmic delivery of active components. Collectively, these findings indicate that EVs can deliver their contents via direct fusion with the plasma membrane or through endocytic pathways followed by fusion with endosomal membranes [39].

**Figure 1:**
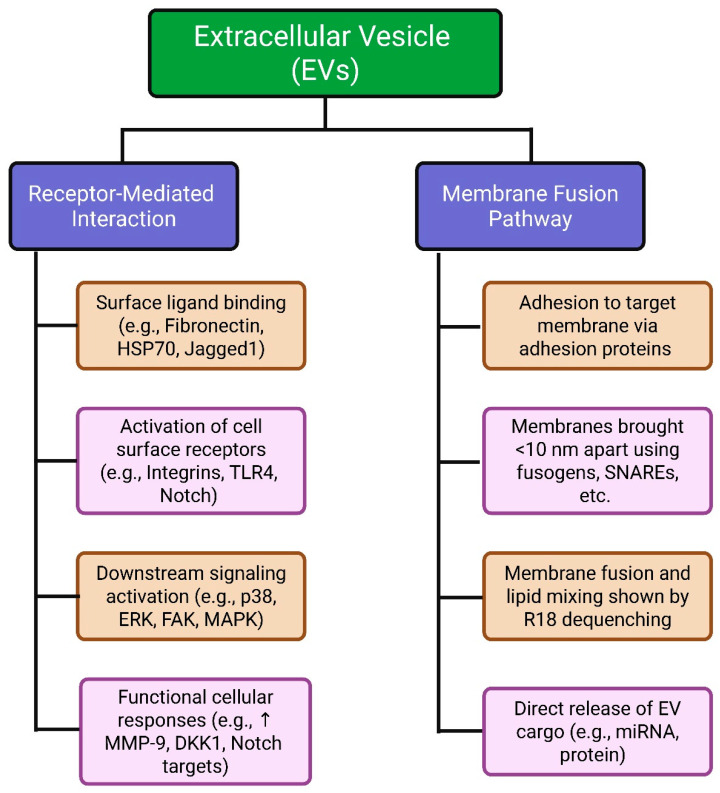
**Mechanisms of EV–cell interaction.** Mechanisms of extracellular vesicle (EV)–cell interaction. EVs interact with target cells via Receptor-Mediated Interactions, involving ligand binding and Membrane Fusion Pathway. EVs: extracellular vesicles; HSP70: heat shock protein 70; TLR4: toll-like receptor 4; p38: p38 mitogen-activated protein kinase; ERK: extracellular signal-regulated kinase; FAK: focal adhesion kinase; MAPK: mitogen-activated protein kinase; MMP-9: matrix metalloproteinase-9; DKK1: Dickkopf-related protein 1; SNAREs: soluble N-ethylmaleimide-sensitive factor attachment protein receptors; R18: octadecyl rhodamine B chloride (fluorescent probe); miRNA: microRNA. *Created in BioRender. Gangadaran, P. (2025) BioRender.com/9uyghiq*.

Despite substantial evidence of EV uptake, the exact mechanisms underlying their internalization remain highly debated. Multiple pathways have been proposed for EV entry, including clathrin-mediated endocytosis (CME), caveolin-mediated endocytosis, phagocytosis, macropinocytosis, and direct fusion with the plasma membrane. Selective protein–protein interactions and lipid raft-dependent mechanisms have also been implicated in facilitating EV uptake. Lipid rafts—cholesterol-rich microdomains within the plasma membrane—organize membrane proteins and signaling molecules and participate in both clathrin- and caveolin-mediated endocytosis, providing platforms for EV binding and internalization [40]. Disruption of lipid rafts generally reduces EV uptake, supporting their functional role. Phagocytosis, predominantly in immune cells, enables internalization of larger EVs via membrane extension and engulfment. Macropinocytosis, a non-specific process, involves membrane ruffling that engulfs extracellular fluid and particles such as EVs into large vesicles known as macropinosomes; EVs may become trapped in these ruffles before internalization. Additionally, EVs can fuse directly with the recipient cell membrane, allowing rapid release of their contents into the cytoplasm. Alternatively, EVs internalized via endocytosis may fuse with the endosomal limiting membrane to release their cargo into the cytosol, where it can exert functional effects. To investigate these mechanisms, researchers commonly use chemical inhibitors to block specific uptake pathways or antibodies to disrupt receptor–ligand interactions. These approaches have been instrumental in delineating the contributions of distinct pathways to EV uptake (Figure 1) [41].

EVs interact with target cells through multiple mechanisms that modulate cellular function and physiology. A principal mechanism is receptor-mediated binding, in which EV surface proteins or ligands—such as fibronectin [42], HSP70 [43], or Jagged1 [44]—bind to corresponding receptors, including integrins, TLR4, and Notch, on target cells. These interactions can initiate signaling cascades such as p38 MAPK, ERK1/2, FAK, and Src family kinases, leading to gene expression changes, including upregulation of MMP-9, DKK1, and various Notch target genes [44]. In parallel, EVs can deliver cargo via direct membrane fusion. This energy-dependent process is typically mediated by specialized proteins, including SNAREs, Rab GTPases, and SM family proteins [45]. Fusion occurs when membranes approach within approximately 10 nm, driven by fusogenic or receptor–ligand interactions and regulated by membrane lipid composition [46].

Experimental support for fusion-mediated delivery includes R18 lipid dequenching assays, which have visualized EV membrane fusion in several systems, such as bone marrow-derived dendritic cells [39], melanoma cells under acidic tumor-like conditions [47], and stromal fibroblasts exposed to keratinocyte-derived EVs [48]. Fusion-mediated miRNA transfer has also been quantified using luciferase reporter assays [39]. Inhibition studies employing botulinum toxin A have demonstrated the involvement of SNARE proteins in EV uptake by breast cancer cells [49]. These diverse pathways underscore the versatility of EVs as mediators of intercellular communication, functioning through both surface receptor engagement and direct cytoplasmic cargo delivery depending on the cellular context [50].

### 2.6. Role of Extracellular Vesicles in Articular Cartilage Homeostasis and Repair

Recent research has highlighted that EVs are critical mediators in articular cartilage homeostasis and regeneration. Chondrocyte-derived EVs contain growth factors, matrix proteins, and regulatory miRNAs that support cartilage matrix synthesis and maintenance [51]. EVs from MSCs promote repair of cartilage defects and exert anti-inflammatory effects by modulating local immune cell responses. Emerging biomaterial strategies deliver EVs directly to cartilage lesions using hydrogel or scaffold systems, which enhance chondrogenesis and inhibit catabolic processes in joint diseases such as osteoarthritis [52]. Therefore, EVs have significant potential as cell-free therapeutics for articular cartilage disorders, complementing their established role in bone regeneration.

## 3. Role of EVs in Osteogenesis

EVs, including exosomes and microvesicles, have emerged as pivotal mediators of intercellular communication within the bone microenvironment. Secreted by a wide range of cell types, these nanoscale vesicles carry bioactive cargo proteins, lipids, mRNAs, and regulatory miRNAs that orchestrate key cellular events in recipient cells. In skeletal tissue engineering, EVs represent a promising cell-free approach capable of recapitulating the regenerative effects of their parent cells [53]. Among EVs, those derived from mesenchymal stem cells (MSCs)—notably from bone marrow, adipose tissue, and dental pulp—are most extensively studied. MSC-derived EVs exhibit potent osteoinductive properties, mediated by cargo such as miR-21, miR-196a, miR-27a, and miR-206, which target inhibitors of osteogenic differentiation, including SMAD7 and DKK1 [54]. Bone marrow MSC-EVs (BM-MSC-EVs), for example, have been shown to induce osteoblast differentiation and enhance bone healing via activation of the Wnt/β-catenin and MAPK pathways, promoting mineral deposition and collagen matrix formation. Similarly, adipose-derived MSC-EVs (AD-MSC-EVs) support osteogenic differentiation and modulate the inflammatory microenvironment, facilitating the repair of osteoporotic or traumatic bone injuries [55]. Dental pulp MSC-EVs deliver angiogenic and neurotrophic cues in addition to osteogenic signals, potentially supporting neovascularized bone tissue engineering [56].

Beyond MSCs, osteoblast-derived EVs (OB-EVs) directly promote matrix mineralization by delivering essential osteogenic proteins such as RUNX2, alkaline phosphatase, and BMP-2, along with calcium and phosphate ions to nucleate hydroxyapatite crystals. These vesicles contribute not only to mineral deposition but also to osteoprogenitor recruitment and differentiation by activating signaling pathways such as TGF-β and Hedgehog [57]. In contrast, osteoclast-derived EVs (OC-EVs), which are primarily involved in bone resorption, display dual regulatory actions. Under pathological conditions like osteoporosis, OC-EVs are enriched in miR-214-3p, which inhibits osteoblast activity by targeting ATF4 and thereby impairs bone formation. Conversely, under homeostatic conditions, OC-EVs may carry RANKL or sphingosine-1-phosphate (S1P), facilitating osteoblast–osteoclast coupling and coordinated bone remodeling [58]. Endothelial cell-derived EVs (EC-EVs) are also critical, particularly in angiogenic–osteogenic coupling, a process essential for the development of functional and vascularized bone grafts. EC-EVs contain VEGF, angiopoietin-1, and miRNAs such as miR-126 and miR-210, which activate PI3K/Akt and ERK pathways in MSCs, thereby promoting both angiogenesis and osteogenesis. This dual function is vital for the integration and survival of engineered bone tissues, especially in ischemic environments [59].

Platelet-derived EVs (P-EVs) also represent a clinically attractive source due to their autologous origin and high concentrations of restorative growth factors such as PDGF, TGF-β, and EGF. These vesicles, which can be isolated from platelet-rich plasma (PRP), have been demonstrated to promote MSC proliferation, migration, and osteogenic differentiation in both in vitro and animal models. Their compatibility with established clinical protocols positions P-EVs as a readily translatable adjunct in orthopedic surgery and bone grafting [60]. Despite these encouraging outcomes, challenges persist in standardizing and scaling EV-based therapies. Variability in EV composition due to donor heterogeneity, inconsistencies in isolation methods (e.g., ultracentrifugation versus size-exclusion chromatography), and the absence of reliable markers to distinguish EV subtypes complicate both research and clinical translation. Furthermore, the long-term biosafety of EVs, particularly concerning off-target effects or potential immunogenicity, warrants comprehensive evaluation in large-scale clinical studies. Nonetheless, innovative strategies such as EV functionalization, cargo engineering, and integration into biomimetic scaffolds are being investigated to optimize targeting, retention, and therapeutic impact. For instance, collagen- or hydroxyapatite-based scaffolds can serve as platforms for sustained EV release and localized activity. Designer EVs engineered to overexpress specific miRNAs or osteoinductive proteins are also being explored as advanced biologics.

### 3.1. EVs Derived from Different Cell Types and Their Effects on Bone Formation

**Figure 2:**
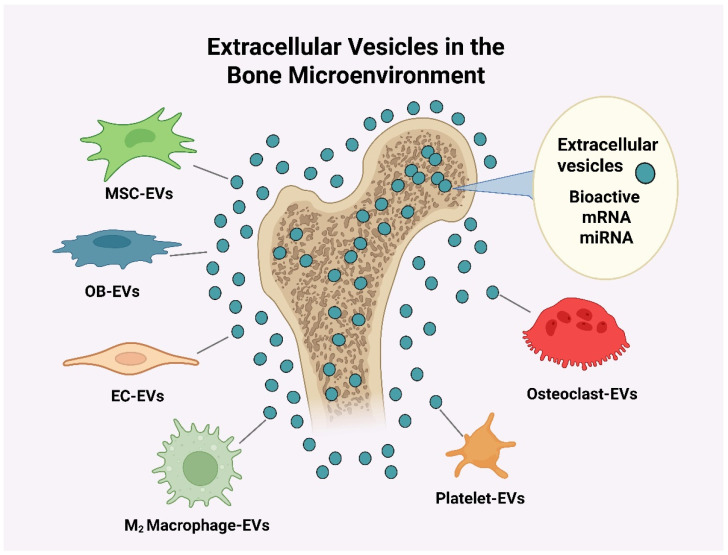
**Illustrations of different types of extracellular vesicles in the bone microenvironment.** EVs: extracellular vesicles; MSC-EVs: mesenchymal stem cell-derived extracellular vesicles; OB-EVs: osteoblast-derived extracellular vesicles; EC-EVs: endothelial cell-derived extracellular vesicles; M_2_ Macrophage-EVs: type 2 macrophage extracellular vesicles; Platelet-EVs: platelet-derived extracellular vesicles; miRNAs: microRNAs; mRNAs: messenger RNAs. *Created in BioRender. Gangadaran, P. (2025) BioRender.com/g8k2s3b*.

MSCs, recognized for their self-renewal and multilineage differentiation capabilities, have become a cornerstone of regenerative medicine, especially in skeletal tissue engineering (Figure 2). Their ability to differentiate into osteoblasts, chondrocytes, and adipocytes establishes them as an ideal cellular source for bone repair. However, accumulating evidence indicates that much of the therapeutic benefit of MSCs is mediated not by direct engraftment or differentiation, but through paracrine signaling, predominantly via EVs such as exosomes and microvesicles. These nanoscale vesicles, typically 30–150 nm in diameter, encapsulate a diverse repertoire of bioactive molecules—including osteoinductive microRNAs (miRNAs), messenger RNAs (mRNAs), proteins, and lipids—that regulate recipient cell behavior in the bone microenvironment. MSC-derived EVs (MSC-EVs) recapitulate many of the beneficial effects of their parent cells, providing a cell-free alternative that avoids the risks of immune rejection, uncontrolled differentiation, or tumorigenesis associated with stem cell therapies. Among the most studied cargo are miRNAs such as miR-196a, miR-27a, miR-29b, and miR-21, which target negative regulators of osteogenesis, including SMAD7, GSK-3β, and PTEN, thereby activating pro-osteogenic signaling pathways such as Wnt/β-catenin, BMP/Smad, and PI3K/Akt cascades [61]. These pathways are essential for osteoblast differentiation, matrix synthesis, and mineralization. In vitro studies have consistently demonstrated that MSC-EVs enhance osteoprogenitor cell proliferation and migration, increase alkaline phosphatase (ALP) activity, upregulate osteogenic markers such as RUNX2 and osteocalcin, and promote calcium deposition and extracellular matrix mineralization.

Bone marrow-derived MSCs (BM-MSCs) are among the most extensively investigated sources of EVs. Their secreted vesicles induce robust osteogenic responses in both two-dimensional and three-dimensional culture systems. Adipose tissue-derived MSCs (AD-MSCs) and umbilical cord-derived MSCs (UC-MSCs) also demonstrate significant osteoinductive capabilities through EV-mediated mechanisms, though their vesicle miRNA profiles and protein contents vary slightly, reflecting tissue-specific expression patterns. Notably, MSC-EVs exhibit not only osteogenic but also immunomodulatory effects, frequently promoting a macrophage phenotype shift from M1 to M2. This transition reduces inflammation and fosters a regenerative microenvironment. In vivo studies using various preclinical models have confirmed the efficacy of MSC-EVs in bone repair. For example, in rodent models of calvarial bone defects, long bone fractures, and osteoporotic lesions, both systemic and local administration of MSC-EVs has led to increased bone volume, trabecular thickness, and biomechanical strength at defect sites [62]. Histological analyses further demonstrate enhanced osteoblast activity and vascularization, supporting the concept that MSC-EVs orchestrate a multifaceted repair process involving osteogenesis, angiogenesis, and immunoregulation.

A key strategy to enhance the therapeutic potential of MSC-EVs involves their integration with biomaterial scaffolds or hydrogels. These platforms mimic the native extracellular matrix and enable the controlled spatial and temporal release of vesicles. Hydrogels composed of natural polymers such as collagen, gelatin, or hyaluronic acid, as well as synthetic polymers like polyethylene glycol (PEG), have been used to encapsulate MSC-EVs for delivery to bone defects. These composite constructs preserve EV bioactivity and improve retention at target sites, thereby enhancing local osteoinduction and tissue integration [63]. Additionally, some scaffold materials synergize with MSC-EVs by providing osteoconductive cues, further promoting bone regeneration. Advanced scaffolds have incorporated osteoinductive peptides, growth factors, or nanoparticles alongside EVs to create multifunctional regenerative platforms.

Recent advances in preconditioning and genetic modification of MSCs have emerged as promising approaches to enhance the osteogenic potential of their EVs. For instance, MSCs pretreated with osteogenic inducers (such as dexamethasone, β-glycerophosphate, or bone morphogenetic proteins) secrete EVs enriched in pro-osteogenic miRNAs and signaling molecules. Similarly, hypoxic or mechanical stimulation of MSCs increases the angiogenic and osteogenic factor content of EVs, improving their efficacy in challenging environments, such as avascular or load-bearing bone defects. Genetic engineering has also been employed to overexpress specific osteoinductive genes or miRNAs within MSCs, tailoring EV cargo for targeted bone healing applications [64].

From a translational perspective, MSC-EVs offer several advantages over conventional cell-based therapies. Their small size and membrane composition confer increased stability and enable traversal of biological barriers, including the endothelial lining and dense bone extracellular matrix. Unlike whole-cell therapies, EVs are less likely to elicit immune responses or form teratomas. Their ease of storage, sterilization, and standardization further supports clinical scalability. However, challenges remain regarding the standardization of isolation techniques, such as ultracentrifugation and size-exclusion chromatography, and the clinical characterization of EVs. Regulatory considerations—including Good Manufacturing Practice (GMP) compliance, production scalability, and batch consistency—must also be addressed before widespread adoption in orthopedic and craniofacial surgery. Moreover, long-term studies on biodistribution, pharmacokinetics, and biosafety are required to define the therapeutic window and potential off-target effects of MSC-EVs. Despite these challenges, current evidence strongly supports the application of MSC-derived EVs as a potent, acellular strategy to enhance osteogenic differentiation and accelerate bone regeneration, representing a promising avenue for next-generation regenerative therapies in skeletal medicine [65].

### 3.2. Molecular Mechanisms Mediated by EVs in Bone Regeneration

Osteoblasts are the principal effector cells responsible for bone matrix synthesis and mineralization. They contribute not only through direct deposition of organic and inorganic matrix components but also by secreting EVs that regulate bone homeostasis and regeneration. Osteoblast-derived EVs (OB-EVs), including exosomes and matrix vesicles, have attracted considerable interest for their active participation in initiating and regulating mineralization of the bone extracellular matrix (ECM) [66]. OB-EVs are nanoscale, membrane-bound vesicles enriched in bioactive proteins, lipids, and nucleic acids that mediate intercellular communication within the osteogenic niche [67]. Key proteins in these vesicles include bone sialoprotein (BSP), osteocalcin (OCN), and tissue non-specific alkaline phosphatase (TNAP), all central to hydroxyapatite (HA) nucleation and crystal growth. TNAP hydrolyzes pyrophosphate (PPi), a potent mineralization inhibitor, into inorganic phosphate (Pi), increasing Pi supersaturation and facilitating HA precipitation on the collagen matrix. BSP and OCN further promote crystal nucleation and anchorage by binding calcium and phosphate ions, establishing the foundational architecture of mineralized bone.

Beyond protein cargo, OB-EVs are enriched with osteoinductive microRNAs (miRNAs) such as miR-143, miR-375, and miR-30d, which regulate expression of osteogenic transcription factors including RUNX2, osterix, and SMAD1 in neighboring osteoprogenitor or MSC populations. miR-143 modulates the ERK5 signaling cascade, affecting osteoblast proliferation and differentiation, while miR-375 influences Wnt and Notch pathways to balance osteogenesis and adipogenesis [68]. The miRNA content of OB-EVs reflects the differentiation and mineralization status of donor osteoblasts, suggesting a dynamic feedback mechanism by which mature osteoblasts influence surrounding progenitor cells and promote coordinated bone formation. OB-EVs may also carry mRNAs encoding bone matrix proteins and enzymes, such as collagen type I and PHOSPHO1, further amplifying osteogenic signaling in target cells [69].

A specialized subclass of OB-EVs, known as matrix vesicles (MVs), is particularly important in bone mineralization. First described over five decades ago, MVs bud directly from the plasma membrane of mature osteoblasts and hypertrophic chondrocytes during endochondral ossification. These vesicles localize within the ECM at mineralization foci and serve as the initial nidus for calcium phosphate deposition. MVs are enriched with annexins (notably annexin A2, A5, and A6), calcium-binding phospholipids, sphingomyelin, and critical enzymes including PHOSPHO1 and TNAP. Annexins mediate calcium ion transport across the vesicle membrane, while PHOSPHO1 hydrolyzes phosphoethanolamine and phosphocholine to generate Pi within the vesicle, creating a high concentration of Ca^2+^ and Pi conducive to HA nucleation. The lipid composition of MVs, including negatively charged phosphatidylserine and sphingomyelin, increases calcium affinity and stabilizes mineral crystal formation.

MVs not only initiate mineralization but also function as delivery vehicles for mineralization regulators and signaling molecules that modulate ECM remodeling. For example, they may contain metalloproteinases and proteoglycans involved in pericellular matrix degradation and reorganization, facilitating integration of new minerals into developing bone tissue. MVs can also promote angiogenesis by carrying pro-angiogenic factors such as VEGF and FGF2, underscoring their role in coupling osteogenesis with neovascularization, which is essential for effective bone regeneration [70].

In vitro, osteoblast-EVs applied to MSCs or osteoprogenitors enhance alkaline phosphatase (ALP) activity, calcium nodule formation, and upregulation of osteogenic genes such as COL1A1, RUNX2, and BGLAP, confirming their capacity to induce osteogenic differentiation. OB-EVs from osteoblasts cultured in osteogenic medium exert a stronger mineralization effect than those from undifferentiated cells, emphasizing the importance of donor cell maturity for vesicle efficacy [71]. Advances in biomaterials have enabled incorporation of OB-EVs into three-dimensional scaffolds for sustained release at bone defect sites. For example, hydroxyapatite or β-tricalcium phosphate (β-TCP) scaffolds functionalized with OB-EVs demonstrate increased mineral deposition and osteointegration in animal models, validating their translational therapeutic potential.

The potential of OB-EVs also extends to modulating pathological mineralization. Dysregulation of vesicle production or function contributes to conditions such as osteomalacia, osteoporosis, and vascular calcification. For instance, defective EV biogenesis or insufficient TNAP or PHOSPHO1 loading can impair HA crystallization, resulting in hypomineralized bone. Conversely, aberrant release of calcifying EVs by vascular smooth muscle cells—phenotypically similar to OB-EVs—has been implicated in ectopic vascular calcification, indicating overlapping mechanisms between physiological and pathological mineralization.

Despite these advances, challenges remain before OB-EVs can be fully translated into clinical applications. Isolation and purification methods, including ultracentrifugation, density gradient centrifugation, and size-exclusion chromatography, require optimization to achieve high yield and purity while preserving vesicle integrity and bioactivity. Additionally, donor variability—such as age, disease status, and metabolic activity—significantly affects EV cargo and efficacy. Standardized protocols for EV characterization, encompassing size distribution, surface marker profiling (e.g., CD63, CD81, ALP), and functional assays, are essential for reproducibility and regulatory compliance. Nonetheless, OB-EVs represent a powerful paradigm in skeletal regenerative medicine. By harnessing the intrinsic mineralization machinery encoded within these vesicles, researchers and clinicians can develop cell-free therapies with improved safety, scalability, and targeted efficacy. Future directions should focus on integrating OB-EVs into composite biomaterials, augmenting their osteoinductive potential through biochemical preconditioning or genetic engineering, and validating their safety in long-term in vivo studies to fully realize their therapeutic promise.

#### 3.2.1. Wnt/β-Catenin Pathway: Activating Osteogenesis

##### Overview of Wnt/β-Catenin Signaling

The Wnt gene was first identified as a homolog of the mouse int-1 gene—originally discovered in mammary tumorigenesis—and the Drosophila wingless gene. Due to the high similarity between these genes and their protein products, the term “Wnt gene” was adopted to encompass both [72].

Wnt signaling comprises both canonical and noncanonical pathways. Noncanonical Wnt signaling operates independently of β-catenin–TCF/LEF (T-cell factor/lymphoid enhancer-binding factor) complexes and includes pathways such as Wnt/Ca^2+^ and the noncanonical Wnt planar cell polarity pathway. These noncanonical pathways regulate processes such as cell polarity and migration [73].

The canonical Wnt pathway, also termed the Wnt/β-catenin pathway, is characterized by β-catenin nuclear translocation and subsequent activation of target genes through TCF/LEF transcription factors. This pathway primarily governs cell proliferation and is also essential for tissue self-renewal in mammals [74].

##### Wnt/β-Catenin Pathway in Osteogenesis

**Figure 3:**
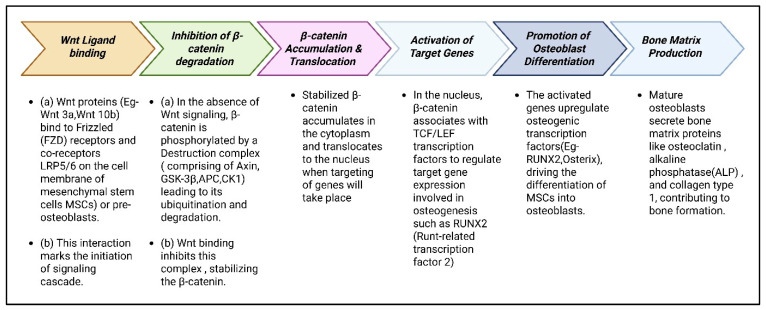
**Schematic representation of the canonical Wnt/β-catenin signaling pathway in osteogenesis.** The binding of Wnt ligands to their receptors inhibits β-catenin degradation, allowing its accumulation and translocation into the nucleus. Nuclear β-catenin activates target gene transcription, which promotes osteoblast differentiation and subsequent bone matrix production. *Created in BioRender. Gangadaran, P. (2025) BioRender.com/y5madsl*.

Wnt signaling initiates osteogenesis by ligand binding, which prevents β-catenin degradation, allowing its accumulation and nuclear translocation. β-catenin then activates target genes, promoting osteoblast differentiation and ultimately bone matrix production (Figure 3) [75].

##### Transcription Factors in Wnt/β-Catenin-Mediated Osteogenesis

Key transcription factors regulate osteogenesis through coordinated mechanisms. β-catenin acts as a co-activator with TCF/LEF to trigger osteogenic gene expression via Wnt signaling. RUNX2, the master regulator, induces osteoblast differentiation and activates bone matrix genes, while Osterix (SP7) functions downstream to mature pre-osteoblasts. Dlx5 enhances RUNX2 expression, and Msx2 promotes early osteoprogenitor proliferation. ATF4 cooperates with RUNX2 to increase matrix protein synthesis. In contrast, Twist1/2 suppresses RUNX2, and their inhibition favors osteogenesis (Table 2) [76].

#### 3.2.2. BMP/TGF-β Pathway

##### Induction of Bone Formation

Bone morphogenetic proteins (BMPs) are signaling molecules within the transforming growth factor-β (TGF-β) superfamily. These proteins promote osteoblast differentiation and bone development through both Smad-dependent and Smad-independent mechanisms [64]. 

BMP/TGF-β pathway: stepwise induction of bone formation:

##### Ligand Synthesis and Activation

BMPs and TGF-β are secreted cytokines of the TGF-β superfamily. They are initially synthesized as inactive precursors that require proteolytic cleavage for activation. BMPs—particularly BMP-2, BMP-4, and BMP-7—are essential for initiating bone formation. In contrast, TGF-β isoforms, including TGF-β1, TGF-β2, and TGF-β3, primarily regulate and promote cell proliferation during bone remodeling [64].

##### Ligand–Receptor Binding and Receptor Complex Formation

Two receptor types are involved, i.e., type I receptors: these are termed activin receptor-like kinases (ALKs), and type II receptors: these function as active serine/threonine kinases. Upon activation, BMP or TGF-β ligands bind to the type II receptor, which then recruits and phosphorylates a type I receptor, resulting in the formation of an active receptor complex [64]. Please refer to Table 3 for easier understanding.

##### SMAD Protein Activation

Canonical pathway: Activated type I receptors phosphorylate receptor-regulated SMADs (R-SMADs). BMPs activate SMAD1, SMAD5, and SMAD8. TGF-β activates SMAD2 and SMAD3. Complex formation: phosphorylated R-SMADs associate with the common mediator SMAD (Co-SMAD), specifically SMAD4, which is required for complex stability and nuclear translocation [77].

##### Nuclear Translocation and Gene Transcription

After the R-SMAD/SMAD4 complex forms, it translocates to the nucleus. Within the nucleus, SMAD complexes function as transcription factors by directly binding DNA or interacting with other transcriptional regulators. They regulate the expression of osteogenic genes that promote the differentiation of progenitor cells into osteoblasts [64]. Please refer to Table 4 to note the role of key osteogenic genes.

##### MSC Differentiation into Osteoblasts

Bone morphogenetic protein (BMP) signaling is essential for directing MSCs toward osteoblastic differentiation. Activation of SMAD pathways and osteogenic transcription factors promotes the transition of MSCs into pre-osteoblasts, which subsequently mature into functional osteoblasts. Transforming growth factor-β (TGF-β), although primarily associated with promoting proliferation, supports early MSC proliferation and extracellular matrix synthesis. However, excessive TGF-β activity can inhibit late-stage osteogenic differentiation [64].

##### Osteoblast Function and Bone Matrix Formation

Matrix production: Mature osteoblasts synthesize osteoid, the unmineralized organic component of the bone extracellular matrix (ECM), which provides the scaffold for mineral deposition. The osteoid is composed chiefly of the following: Type I collagen—constitutes approximately 90% of the organic matrix and confers tensile strength. Osteocalcin—binds calcium and modulates mineral maturation. Osteonectin—connects collagen with hydroxyapatite and facilitates mineralization. Bone sialoprotein—initiates mineral deposition. Proteoglycans—regulate water content, ion transport, and collagen fiber organization [78]. Mineralization—following osteoid deposition, osteoblasts initiate matrix mineralization by depositing calcium phosphate as hydroxyapatite crystals (Ca_10_(PO_4_)_6_(OH)_2_), which impart bone rigidity and hardness [79].

##### Osteoblast Fate and Bone Maturation

After matrix formation, osteoblasts follow one of three possible fates, as summarized in the following Table 5 [78].

##### Negative Regulation and Feedback Loops

Please refer to Table 6 to know in detail different negative regulators and their mechanisms.

#### 3.2.3. PI3K/Akt and MAPK Signaling: Enhancing Cell Survival and Proliferation

##### The Phosphoinositide 3-Kinase (PI3K/Akt) Signaling Pathway

The PI3K/Akt pathway is central to the regulation of cell survival, proliferation, and metabolism.

Overview of the PI3K/Akt signaling pathway: Ligand binding and receptor activation: Extracellular growth factors, including insulin, insulin-like growth factor 1 (IGF-1), and epidermal growth factor (EGF), bind to their respective receptor tyrosine kinases (RTKs) on the cell surface. This interaction induces receptor dimerization and autophosphorylation of specific cytoplasmic tyrosine residues, generating docking sites for downstream signaling molecules [80]. Recruitment and activation of PI3K: Phosphorylated RTKs recruit and activate class IA phosphoinositide 3-kinases (PI3Ks) through the SH2 domain-containing regulatory subunit (p85). The p85 subunit binds to phosphorylated tyrosine residues on RTKs, positioning the catalytic subunit (p110) at the plasma membrane for activation [80]. Generation of PIP3: Activated PI3K phosphorylates the 3′-hydroxyl group of phosphatidylinositol 4,5-bisphosphate (PIP2), converting it into phosphatidylinositol 3,4,5-trisphosphate (PIP3). PIP3 acts as a secondary messenger, accumulating at the inner leaflet of the plasma membrane to recruit proteins with pleckstrin homology (PH) domains [80]. Recruitment of Akt and PDK1 to the membrane: PIP3 accumulation at the plasma membrane recruits Akt (protein kinase B) and phosphoinositide-dependent kinase-1 (PDK1) via their PH domains. Membrane colocalization is required for subsequent Akt activation [80]. Activation of Akt: Akt is activated by phosphorylation at two key residues: threonine 308 (Thr 308) in the activation loop by PDK1, and serine 473 (Ser 473) in the hydrophobic motif by PDK2, a component of the mTORC2 complex. This dual phosphorylation fully activates Akt, enabling phosphorylation of numerous downstream substrates [80]. Downstream effects of Akt activation: Activated Akt phosphorylates a broad spectrum of substrates involved in cell survival and proliferation. Akt activation triggers diverse biological responses through modulation of these targets [80]. Please refer to Table 7 for the summary of the Akt activation downstream effects and key transcription factors.

##### MAPK Signaling Pathway

The MAPK (mitogen-activated protein kinase), also known as ERK (extracellular signal-regulated kinase), cascade is a key regulator of cell survival and proliferation. This pathway operates through a precisely controlled sequence of phosphorylation events.

Pathway of MAPK signaling—Receptor activation: Receptor tyrosine kinases (RTKs) or G protein-coupled receptors (GPCRs) are activated upon binding mitogenic ligands, such as growth factors including EGF and PDGF. Ligand engagement triggers receptor dimerization and autophosphorylation, generating docking sites for adaptor proteins such as Grb2. Grb2 then recruits SOS, a guanine nucleotide exchange factor (GEF), which activates RAS by facilitating the exchange of GDP for GTP [82]. 

##### Three-Tiered Kinase Cascade

Table 8 shows the core MAPK module, which involves sequential phosphorylation.

##### Nuclear Translocation and Transcriptional Regulation

Upon activation, ERK1/2 can either remain in the cytoplasm to phosphorylate cytosolic substrates or translocate into the nucleus, where it modulates transcription factor activity. Please refer to Table 9 to understand key transcription factors and their mechanisms.

##### Gene Expression and Proliferation Outcomes

MAPK-activated transcription factors bind specific DNA sequences, such as AP-1 or CRE sites, within target gene promoters. This binding initiates the transcription of genes that promote cell survival and proliferation. Key genes regulated in this manner include the following: Cyclin D1: c-FOS/c-JUN (AP-1) binds TPA response elements (TREs) in the cyclin D1 promoter, upregulating its expression and driving the G1/S cell cycle transition. Bcl-2, Mcl-1: CREB activates Bcl-2 transcription, enhancing cell survival by inhibiting mitochondrial apoptosis. c-Myc: promotes cell proliferation and survival [83].

### 3.3. Endothelial Cell-Derived EVs: Angiogenesis and Osteogenesis Coupling

Regeneration of bone tissue, particularly in large segmental defects or ischemic environments, requires not only robust osteogenesis but also the formation of a well-organized vascular network to support cellular viability and metabolic activity. Vascular endothelial cells (ECs), which line the inner surface of blood vessels, are essential for bone repair through their dual role in initiating and sustaining angiogenesis and coordinating osteogenesis. A principal mechanism by which ECs influence bone healing is the secretion of EVs, including exosomes and microvesicles, collectively termed EC-derived EVs (EC-EVs). These vesicles encapsulate diverse bioactive molecules—proteins, lipids, mRNAs, and miRNAs—that mediate intercellular signaling along the endothelial–osteogenic axis. EC-EVs are increasingly recognized for their ability to couple angiogenesis and osteogenesis, a process critical for the survival, proliferation, and differentiation of osteoprogenitor cells in regenerative bone environments [86].

Key angiogenic mediators within EC-EVs include vascular endothelial growth factor (VEGF), angiopoietin-1 (Ang1), and fibroblast growth factor-2 (FGF2). These molecules drive endothelial cell proliferation, migration, and capillary tube formation, while delivery via EVs ensures spatially restricted and temporally controlled release within the local tissue microenvironment. VEGF, in particular, acts by binding to VEGFR-2 on endothelial cells, triggering downstream phosphorylation cascades that promote angiogenic sprouting and vessel maturation. Concurrently, these factors exert indirect osteogenic effects by establishing a perfused niche favorable for bone matrix deposition and remodeling. The osteoinductive capacity of EC-EVs is further supported by their miRNA cargo, notably miR-126 and miR-210. miR-126 enhances endothelial cell proliferation and tube formation via PI3K/Akt and MAPK/ERK signaling, and also modulates osteogenic marker expression, such as RUNX2 and osteocalcin, in MSCs through paracrine interaction [62]. miR-210, termed the “hypoxamir,” is pivotal for cell survival and angiogenesis under hypoxic conditions, and enhances MSC osteogenic differentiation by targeting EFNA3 and activating HIF-1α pathways.

This angiogenic–osteogenic coupling mediated by EC-EVs is especially significant in large- or critical-sized bone defects, where vascularization is often the rate-limiting factor for graft survival and integration. In ischemic bone defects or impaired healing associated with diabetes or aging, insufficient blood supply leads to cellular apoptosis and compromised osteogenesis. EC-EVs, through their dual functionality, help overcome these limitations by simultaneously promoting endothelial progenitor cell (EPC) recruitment and activation, and stimulating osteogenic differentiation of local or transplanted MSCs. In vitro studies demonstrate that EC-EVs enhance MSC migration, proliferation, and expression of osteogenic genes such as ALP, COL1A1, and BGLAP under both normoxic and hypoxic conditions [78]. Further, EC-EVs increase capillary-like structure formation by EPCs on Matrigel, indicating pronounced angiogenic efficacy. When co-cultured with osteoblasts or pre-osteoblasts, EC-EVs upregulate transcription factors essential for osteoblast differentiation and matrix mineralization, underscoring their osteoinductive potential [87].

EC-EVs also play a significant role in matrix remodeling. Proteomic analyses reveal the presence of matrix metalloproteinases (MMPs), particularly MMP-2 and MMP-9, which degrade basement membrane components to facilitate neovascularization and mesenchymal cell invasion. Additionally, EC-EVs carry integrins and adhesion molecules such as ICAM-1 and VCAM-1, aiding in vesicle tethering to target cells and modulation of intracellular signaling. These adhesive interactions may promote selective uptake by osteoprogenitor cells, directing osteogenic cues to repair sites. Temporal synchronization of vascular and bone development is further supported by evidence that EC-EVs can modulate Notch and BMP signaling pathways, both critical for vascular branching and osteoblast lineage commitment. Collectively, these findings highlight the potential of EC-EVs as multifunctional vehicles orchestrating the complex biological interactions required for effective bone regeneration.

Emerging biomaterial strategies aim to harness the regenerative potential of EC-EVs by incorporating them into engineered scaffolds. For example, collagen, gelatin, and poly(lactic-co-glycolic acid) (PLGA)-based scaffolds loaded with EC-EVs enable sustained release and localized action at bone defect sites. In vivo models show that EC-EV-functionalized scaffolds enhance vascular density, improve callus formation, and increase expression of osteogenic and angiogenic markers compared to controls. The proximity of newly formed blood vessels to mineralized bone tissue further supports the synergy between angiogenesis and osteogenesis mediated by EC-EVs. Additionally, these constructs reduce inflammatory infiltration and promote better integration with host tissue, emphasizing their immunomodulatory benefits [88].

Despite these advances, clinical translation of EC-EV-based therapies requires standardized protocols for vesicle isolation, quantification, and quality control. Ultracentrifugation, tangential flow filtration, and size-exclusion chromatography are commonly used techniques, each with trade-offs between yield, purity, and scalability. Furthermore, heterogeneity among EC sources—including human umbilical vein endothelial cells (HUVECs), microvascular endothelial cells, and induced pluripotent stem cell-derived ECs—can result in variability in EV composition and functional potency [89]. Establishing well-characterized and reproducible EV preparations is thus essential for consistent therapeutic outcomes. In addition, long-term biodistribution, safety, and immunogenicity of EC-EVs in humans remain active areas of investigation.

### 3.4. Macrophage-Derived EVs: Modulating Inflammatory Responses for Bone Healing

Bone repair proceeds through sequential inflammatory, reparative, and remodeling phases, each controlled by a tightly regulated network of cellular and molecular mediators. Among the central regulators are macrophages, versatile immune cells with the capacity to orchestrate tissue regeneration through phenotypic plasticity. Macrophages adopt functionally distinct polarization states, primarily as classically activated M1 macrophages, which secrete pro-inflammatory cytokines to initiate repair, or alternatively activated M2 macrophages, which mediate inflammation resolution and tissue remodeling [90]. Increasing evidence indicates that the regenerative potential of macrophages depends not only on soluble cytokines and surface receptors but also on their secretion of EVs, which serve as precisely regulated paracrine effectors in the bone healing environment [91]. Macrophage-derived EVs (Mφ-EVs) carry diverse biologically active cargo—including microRNAs (miRNAs), anti-inflammatory cytokines, lipids, and functional proteins—that collectively modulate immune responses and bone regeneration in a context-dependent manner [92].

M2 macrophage-derived EVs (M2-EVs) have emerged as potent immunomodulatory and osteoregenerative agents, owing to their enriched content of anti-inflammatory molecules and regenerative cues. These EVs contain cytokines such as interleukin-10 (IL-10) and transforming growth factor-beta (TGF-β), both crucial for suppressing inflammatory cascades and facilitating tissue regeneration. M2-EVs are also enriched in miRNAs such as miR-223, miR-146a, and members of the let-7 family, which inhibit nuclear factor-κB (NF-κB) signaling and downregulate pro-inflammatory mediators, including TNF-α, IL-6, and IL-1β [56]. These vesicles reprogram the local immune microenvironment, reducing excessive inflammation that would otherwise impair osteoblast differentiation and matrix deposition. In vitro studies demonstrate that M2-EVs enhance osteogenic differentiation of MSCs, as indicated by upregulation of markers such as alkaline phosphatase (ALP), osteopontin (OPN), and runt-related transcription factor 2 (RUNX2). Simultaneously, these EVs promote endothelial cell proliferation and tube formation, supporting the coupled processes of angiogenesis and osteogenesis essential for bone regeneration [93].

In vivo, M2-EVs have produced favorable outcomes in bone defect models, where administration resulted in increased bone volume, trabecular thickness, and mechanical strength. Scaffold-based delivery systems incorporating M2-EVs significantly accelerated defect closure in rat calvarial and femoral models, accompanied by increased osteogenic and vasculogenic gene expression in host tissues [94]. The immunomodulatory effects of M2-EVs are especially valuable in aged or diabetic bone, where chronic inflammation and immune dysregulation impede effective repair. By promoting a shift toward a pro-regenerative M2 phenotype, M2-EVs foster a microenvironment conducive to osteoprogenitor recruitment, vascular ingrowth, and matrix mineralization [95].

Conversely, M1 macrophage-derived EVs (M1-EVs), although mainly associated with inflammation propagation, also display paradoxical pro-regenerative functions under specific temporal and spatial conditions. These vesicles are rich in pro-inflammatory cytokines such as IL-1β and TNF-α, as well as miRNAs including miR-155 and miR-21, which activate toll-like receptor (TLR) signaling and induce NF-κB-mediated transcription of inflammatory mediators. While chronic or excessive exposure to M1-EVs can impair osteogenesis by promoting osteoclastogenesis and matrix degradation, a transient M1-EV signal during early injury stages is necessary for priming regeneration. Specifically, M1-EVs recruit MSCs to the injury site and activate early osteogenic signaling cascades, such as the MAPK and Wnt/β-catenin pathways, thus initiating tissue regeneration [96]. Additionally, M1-EVs stimulate expression of vascular endothelial growth factor (VEGF) and basic fibroblast growth factor (bFGF), supporting neovascularization and tissue oxygenation [94].

The dynamic interplay between M1- and M2-macrophage-derived EVs underscores the critical role of temporal modulation in achieving optimal bone regeneration. Early-phase M1-EVs initiate inflammation and mobilize progenitor cells, whereas late-phase M2-EVs promote inflammation resolution and direct tissue reconstruction. This sequential polarization and EV-mediated signaling exemplify the biphasic nature of bone healing, in which a tightly regulated transition from inflammatory to reparative phases is essential. Disruption of this equilibrium, as seen in chronic inflammation, infection, or aging, leads to impaired fracture healing, non-union, or fibrotic scarring. Consequently, leveraging the immunoregulatory functions of Mφ-EVs emerges as a promising therapeutic strategy to modulate immune responses and enhance bone repair [97].

Biomaterial platforms engineered for controlled Mφ-EV delivery have broadened therapeutic possibilities. Hydrogels, electrospun scaffolds, and 3D-printed constructs incorporating Mφ-EVs achieve spatiotemporal release profiles that mimic physiological macrophage dynamics during bone repair. For instance, dual-delivery systems that sequentially release M1- and M2-EVs have been developed to replicate the endogenous healing timeline, resulting in synergistic enhancements in neovascularization, collagen deposition, and mineralization [98]. These hybrid systems exploit the initial pro-inflammatory stimulus from M1-EVs to drive cellular recruitment and immune activation, followed by the immunosuppressive and regenerative actions of M2-EVs to consolidate repair and restore homeostasis.

Despite these advances, significant challenges remain in translating Mφ-EV-based therapies to clinical practice. Standardization of EV isolation methods—including ultracentrifugation, tangential flow filtration, and size-exclusion chromatography—is required to ensure reproducibility and scalability. Additionally, inter-donor variability, influenced by macrophage sources, activation stimuli, and culture conditions, contributes to heterogeneity in EV composition and function. Rigorous characterization using nanoparticle tracking analysis, Western blotting, and RNA sequencing is essential for quality assurance. Furthermore, the long-term safety profile, immunogenicity, and biodistribution of Mφ-EVs must be systematically evaluated in preclinical and clinical models.

## 4. Therapeutic Applications of EVs in Bone Regeneration

EVs are nanoscale vesicles secreted by cells that play pivotal roles in intercellular communication by transporting bioactive molecules, including proteins, lipids, and nucleic acids. Their potential in bone tissue regeneration, particularly fracture healing, has attracted increasing attention due to their ability to influence multiple stages of the healing process.

### 4.1. EVs for Fracture Healing

Fracture healing is a complex process encompassing inflammation, soft callus formation, hard callus development, and bone remodeling. Each stage is modulated by EVs. An EV-mediated cross-talk constitutes a major mode of cell–cell communication, enabling the transfer of signaling molecules that coordinate cellular activities. These interactions synchronize functions within cell populations and facilitate coordination between different cell types. Upon activation, osteoclasts, platelets, neutrophils, macrophages, osteoblasts, and mesenchymal stem cells all release EVs, which promote fracture healing. Moreover, evidence suggests that EVs may be used for diagnosing and treating delayed fracture healing and are involved in the pathophysiology of fracture healing disturbances [99]. Exosomes derived from mesenchymal stromal cells (MSCs) have demonstrated greater efficacy than parental cells in improving the myofibroblastic phenotype [100]. Unlike traditional molecular mechanisms and signal transduction pathways, the selective encapsulation of exosomes enhances their therapeutic potential, providing a functional rationale for their clinical application. In addition, exosomes possess unique surface molecular configurations acquired from parent cells and through electrostatic interactions with extracellular components, conferring a “homing ability” that facilitates targeted delivery [101].

#### 4.1.1. Facilitating Bone Regeneration

EVs, particularly those from mesenchymal stem cells (MSCs), promote osteoblast proliferation, differentiation, and mineralization—key events in new bone formation. They deliver osteogenic signals and growth factors to fracture sites, accelerating the healing cascade. For example, EVs from TGF-β1-stimulated MSCs have shown increased efficacy in facilitating fracture healing in animal models by delivering LRP5.

#### 4.1.2. Regulation of the Immune Response

The inflammatory phase after fracture is critical yet must be tightly regulated. EVs serve as immunomodulatory messengers that influence immune cell activity, including macrophage polarization. Certain EVs induce a shift toward the anti-inflammatory M2 macrophage phenotype, which is essential for tissue repair and regeneration. For example, exosomes derived from Schwann cells induce osteogenesis in BMSCs by polarizing macrophages toward the M2 phenotype.

#### 4.1.3. Enhancement of Vascularization (Angiogenesis)

Neovascularization is necessary for delivering oxygen and nutrients to the fracture site and supporting bone cell function. EVs can stimulate angiogenesis by promoting endothelial cell proliferation, migration, and tube formation. For instance, EVs from umbilical cord mesenchymal stem cells expressing HIF-1α induce VEGF expression, enhance angiogenesis, and improve fracture healing [102].

#### 4.1.4. Comparative Effects of EVs, Demineralized Bone Matrix, and Decellularized Bone Matrix on Bone Regeneration

Demineralized bone matrix (DBM) and decellularized bone matrix (DCBM) are widely used biomaterials for bone regeneration, serving as osteoconductive scaffolds and containing native growth factors essential for bone healing. DBM provides both a structural framework and intrinsic osteoinductivity, primarily due to bone morphogenetic proteins (BMPs) and its particulate nature, although efficacy can be influenced by donor variability and formulation [103]. DCBM retains the extracellular collagenous architecture and supports robust cell adhesion and proliferation, but may require further functionalization to achieve high osteoinductive potential. In contrast, extracellular vesicles (EVs) promote bone regeneration by transferring miRNAs and other bioactive cargo to target cells, enhancing osteogenesis, angiogenesis, and modulating immune responses [5]. Recent bioengineering advances allow integration of EVs with bone matrices, such as EV-functionalized DBM or DCBM, resulting in scaffolds with synergistic pro-bone and pro-angiogenic activities. Thus, while DBM and DCBM primarily act as physical and biochemical scaffolds, EVs provide dynamic molecular cues, suggesting that combination strategies may maximize regenerative outcomes by harnessing both structural and regulatory factors [104].

#### 4.1.5. Advantages over Conventional Therapies

Compared with cell-based therapies, EVs offer several advantages, including lower immunogenicity, greater stability, and ease of storage and sterilization. These properties position EVs as a promising cell-free therapeutic modality for bone repair.

#### 4.1.6. Mechanisms of Action

EVs mediate fracture healing through multiple mechanisms:

➢ Cargo delivery: EVs deliver proteins, lipids, mRNAs, and miRNAs that modulate recipient cell behavior, promoting osteogenic differentiation, angiogenesis, and immunomodulation.
➢ Signaling pathway modulation: EVs can activate or suppress key signaling pathways in target cells involved in bone repair, such as Wnt/β-catenin, TGF-β/SMAD, and PI3K/Akt.
➢ Extracellular matrix interaction: Certain EV components interact directly with the extracellular matrix, modifying its structure and facilitating mineralization.

#### 4.1.7. Sources of EVs for Fracture Healing

EVs derived from various cell types have demonstrated promise in promoting fracture healing:

➢ Bone marrow mesenchymal stem cells (BMSCs): BMSC-derived EVs are extensively studied for their osteogenic and immunomodulatory properties.
➢ Adipose-derived stem cells (ASCs): ASC-derived EVs can enhance the osteogenic potential of BMSCs and support bone regeneration.
➢ Umbilical cord mesenchymal stem cells (uMSCs): EVs from uMSCs exhibit pro-angiogenic and fracture healing activities.
➢ Macrophages: Macrophage-derived EVs, particularly those from the M2 phenotype, participate in inflammation regulation and bone repair [102,105].
➢ Exosomes derived from endothelial progenitor cells (EPCs) accelerate bone repair by promoting differentiation and recruitment of osteoclast precursors via LncRNA-MALAT1.

#### 4.1.8. Delivery Strategies

To enhance the therapeutic efficacy of EVs in fracture healing, several delivery approaches that are under investigation are as follows:

➢ Local injection: Direct injection of EVs into the fracture site enables targeted delivery and higher local concentrations.
➢ Scaffold integration: Incorporating EVs into biomaterial scaffolds provides sustained release and augments bone regeneration at the defect site.
➢ Systemic administration: Although less targeted, systemic delivery may be beneficial in cases involving systemic inflammation.

EV-based therapies hold significant potential to enhance bone regeneration and promote fracture healing. Their multifaceted modulation of the healing process, combined with advantages over traditional cell therapies, makes them a compelling focus for future clinical translation in orthopedics. Further studies optimizing EV sources, dosages, and delivery routes and elucidating their mechanisms of action, are essential to advance clinical application for bone fracture treatment.

### 4.2. EVs in Osteoporosis Therapy

Osteoporosis is a prevalent disease characterized by systemic loss of bone mass and microarchitectural deterioration, resulting in greater susceptibility to fragility fractures. The complex mechanisms regulating bone homeostasis are significantly influenced by EVs. EVs have also gained considerable interest as potential therapeutic agents for osteoporosis, a condition marked by reduced bone mineral density and increased fracture risk due to disrupted bone remodeling. As nanoscale vesicles secreted by cells, EVs transport diverse bioactive molecules and modulate both recipient cells and the local microenvironment [106].

The therapeutic utility of EVs in osteoporosis lies in their ability to regulate bone homeostasis by modulating bone formation (osteogenesis) and bone resorption (osteoclastogenesis):

#### 4.2.1. Promoting Bone Formation (Osteogenesis)

○ EVs derived from osteoblasts, endothelial cells, myocytes, and especially mesenchymal stem cells (MSCs) deliver proteins (including BMPs, OPG, CLEC11A, CTHRC1), microRNAs (miRNAs), and other factors that stimulate osteoblast proliferation, differentiation, and matrix mineralization. For example, MSC-derived EVs can upregulate β-catenin, a key signaling molecule in osteogenesis.
○ EVs from multiple sources, such as osteoblasts, osteoclasts, and MSCs, regulate the balance between bone formation and resorption, thereby affecting the development and progression of osteoporosis. Additionally, EVs can serve as drug carriers to enhance drug targeting and bioavailability in bone tissue, offering a promising strategy for osteoporosis diagnosis and therapy.
○ Specific miRNAs transported by EVs, such as miR-186 and miR-150-3p, have been shown to promote osteogenic differentiation. Conversely, miRNAs like miR-206 and miR-31 suppress osteogenesis, highlighting the context-dependent effects of EVs. Similarly, certain long non-coding RNAs (lncRNAs), including lncRNA-MALAT1, may inhibit osteogenic differentiation.
○ Engineered EVs loaded with osteogenic factors or miRNAs can further augment bone formation.

#### 4.2.2. Inhibiting Bone Resorption (Osteoclastogenesis)

○ EVs can inhibit both the activity and formation of osteoclasts, which are responsible for bone resorption. For example, EVs derived from adipose-derived stem cells (ADSCs) containing miR-21-5p suppress osteoclast differentiation.
○ EVs from mesenchymal stem cells (MSCs) can disrupt the RANKL/RANK pathway—a key axis in osteoclastogenesis—thereby reducing bone resorption. Some EVs also deliver osteoprotegerin (OPG), a decoy receptor for RANKL that blocks osteoclast activation [107].
○ EVs loaded with specific osteoclastogenesis inhibitors, such as antagomiR-31a-5p, have been shown to reduce bone loss.

#### 4.2.3. Modulating the Bone Microenvironment and Inflammation

○ Osteoporosis is typically associated with a pro-inflammatory milieu that promotes bone resorption. EVs, particularly those derived from MSCs, possess immunomodulatory properties. They can modulate the balance between pro- and anti-inflammatory signaling, indirectly supporting bone health.
○ MSC-derived EVs also induce angiogenesis—the formation of new blood vessels—which is essential for nutrient and oxygen supply to bone tissue and supports bone regeneration.

#### 4.2.4. Sources of EVs for the Treatment of Osteoporosis

Recent advances have revealed the complex functions of non-coding RNAs (microRNAs) and long non-coding RNAs in epigenetic regulation. Substantial evidence indicates that non-coding RNAs are transferred between cells and tissues via EVs, enabling delivery to distant targets and systemic effects. Resident bone cells, including osteoclasts, osteoblasts, osteocytes, and endothelial cells, are strictly regulated by non-coding RNAs, many of which are secreted to neighboring cells through EVs, contributing to pathological conditions. Consequently, researchers are exploring the use of EVs as theranostic agents for osteoporosis treatment [108].

#### 4.2.5. Cell Sources for Therapeutic EVs in Osteoporosis

✓ Mesenchymal stem cells (MSCs): EVs from bone marrow (BMSCs), adipose tissue (ADSCs), and umbilical cord (UCMSCs). MSCs are the most extensively studied due to their multipotent differentiation capacity and paracrine signaling, which enhance bone regeneration and suppress bone loss.
✓ Osteoblasts: Osteoblast-derived EVs may directly deliver factors that promote bone matrix deposition and regulate osteoclast activity.
✓ Endothelial cells: EVs from endothelial cells can stimulate angiogenesis, crucial for maintaining bone integrity.
✓ Immune cells (e.g., macrophages): The effects of immune cell–derived EVs in osteoporosis are complex and context-dependent; EVs from M2-polarized macrophages are generally associated with pro-regenerative activities.
✓ Urine-derived stem cells (USCs): EVs from USCs have demonstrated the capacity to promote bone formation and inhibit osteoclasts [96].

#### 4.2.6. Advantages of EV Therapy for Osteoporosis

➢ Cell-free approach: Lower risk of complications related to cell transplantation, such as immune rejection and ectopic tissue formation.
➢ Improved safety profile: Typically less immunogenic and safer than cell-based therapies.
➢ Potential for targeted delivery: EVs can be engineered to target specific cell types or tissues within the bone microenvironment.
➢ Stability and storage: EVs exhibit greater stability and are easier to store than live cells.
➢ Customizable cargo: The therapeutic properties of EVs can be optimized by preconditioning donor cells or directly loading EVs with drugs or genetic material.

### 4.3. EV-Based Approaches in Bone Tissue Engineering

EVs have emerged as promising tools in bone tissue engineering by recapitulating the regenerative effects of parent cells while circumventing limitations of cell-based therapies. Incorporating EVs into biomaterials produces bioactive scaffolds that promote bone regeneration [109]. For example, scaffolds composed of collagen, hydroxyapatite, or poly(lactic-co-glycolic acid) (PLGA) loaded with MSC-EVs enhance osteoblast proliferation, differentiation, and matrix mineralization by delivering osteogenic cargo such as miR-196a and BMP-2. These scaffolds provide structural support, mimic the bone extracellular matrix, and enable controlled EV release, improving retention and bioactivity at defect sites [110]. In vivo studies have shown that EV-functionalized scaffolds increase bone volume, trabecular thickness, and vascularization in critical-sized defects. Furthermore, 3D bioprinting enables precise spatial distribution of EVs within constructs, optimizing therapeutic efficacy. Key challenges include maximizing EV loading efficiency, ensuring scaffold biocompatibility, and scaling up production for clinical translation [111].

### 4.4. Comparison of EV Therapy with Traditional Bone Grafting

EV-based therapies offer multiple advantages over conventional bone grafting methods, including autografts, allografts, and synthetic substitutes. While autografts are osteoinductive, their use is limited by donor site morbidity and availability [112]. Allografts present risks of immune rejection and disease transmission, and synthetic grafts often lack bioactivity. In contrast, EV therapies provide a cell-free alternative that reduces immunogenicity and eliminates risks of uncontrolled cell differentiation or tumorigenesis. EVs deliver targeted osteogenic and angiogenic cues, enhancing bone formation and vascularization without the need for live cells. They are also easier to store, sterilize, and standardize compared to grafts [113]. However, EV therapies face challenges related to scalability, cost, and long-term safety compared to established grafting techniques. Although autografts remain the gold standard for large defects, EV-functionalized scaffolds have demonstrated comparable efficacy in preclinical models, indicating potential as complementary or alternative strategies [109]. Please refer to Table 10 for better understanding. Further clinical trials are necessary to confirm their efficacy relative to traditional approaches.

## 5. Engineering and Enhancement of EVs for Osteogenic Therapy

Recent advances in EV engineering have significantly improved their osteogenic potential. Two main strategies are employed, i.e., modifying the intrinsic content of EVs via genetic or chemical manipulation of source cells and loading bone-stimulating agents into EVs after isolation. These approaches address the limitations of natural EVs, enabling the development of more efficient and targeted nanotherapies for bone repair.

### 5.1. Genetic and Chemical Modifications to Enhance EV Cargo

Genetic engineering of parent cells is an effective method for enriching EV cargo with specific nucleic acids, proteins, or signaling molecules involved in bone formation. Overexpression of osteogenic transcription factors such as BMP2 in donor cells results in EVs enriched with pro-osteogenic components, promoting osteoblastic differentiation and bone healing in vivo [4,115]. For example, BMP2-overexpressing MSCs produce EVs that activate the Smad1/5/8 pathway and promote mineral deposition in both in vitro and animal models [115]. Additionally, MSCs engineered to express bone-targeting ligands like SDF1 generate EVs with enhanced chemotactic attraction to bone tissue, improving localization and therapeutic potential.

Recent studies demonstrate that genetically modified MSCs expressing osteogenic microRNAs—including miR-21, miR-26a, and miR-29b—release EVs capable of modulating key bone formation pathways. These EVs stimulate osteoblast differentiation by activating PI3K/Akt, Wnt/β-catenin, and MAPK signaling, thereby enhancing bone repair.

EVs bearing miR-29b have been shown to suppress osteoclastogenesis genes and upregulate RUNX2 expression in target cells, promoting bone formation while inhibiting bone resorption.

Circular RNAs (circRNAs) have also been utilized to engineer EVs; for instance, circLPAR1- and circGNAQ-enriched EVs from MSCs enhance osteogenic differentiation by sequestering miR-31-5p and miR-539-5p, both of which inhibit osteogenesis [102,103]. This system allows for durable gene regulation due to the stability and structural integrity of circRNAs. In addition, EVs derived from circRNA-transduced MSCs have shown improved therapeutic efficacy in critical-sized bone defect models [116].

Another strategy involves preconditioning donor cells with small molecules such as dimethyloxalylglycine (DMOG), which mimics hypoxia and upregulates hypoxia-inducible factor 1-α (HIF-1α), thereby enhancing the angiogenic and osteogenic properties of EVs via the AKT/mTOR pathway. DMOG-treated MSCs secrete EVs that significantly increase vascularization and bone regeneration, providing synergistic effects critical for repairing large defects.

Beyond nucleic acid cargo, protein engineering approaches have been explored. For example, fusing lysosome-associated membrane protein 2b (Lamp2b) with bone-targeting peptides such as RGD enables their display on EV surfaces, facilitating preferential uptake by osteoblasts [115,117]. Additionally, chemical modifications—including click chemistry and bioconjugation—allow EVs to be functionalized with alendronate or hydroxyapatite-binding groups, further increasing bone specificity [117].

Together, these methods underscore the versatility of genetic and chemical engineering in customizing EVs to improve bone targeting and regenerative efficacy, addressing key challenges such as targeting specificity, osteoinductive potency, and therapeutic consistency.

### 5.2. Loading of Osteoinductive Molecules into EVs

In addition to modifying donor cells, EVs can be directly loaded with osteoinductive agents after isolation using physical or chemical techniques. Methods such as electroporation, sonication, freeze–thaw cycles, or incubation efficiently introduce small molecules, proteins, or nucleic acids into EVs [118]. For example, direct loading of BMP2 protein or mRNA into EVs greatly enhances their osteoinductive potential in bone defect models; BMP2-loaded EVs show increased mineral deposition and ALP activity in vitro compared to unmodified EVs [119].

Therapeutic microRNAs such as miR-26a, miR-29b, and miR-218 have been introduced into isolated EVs via electroporation, resulting in increased osteogenesis and angiogenesis upon delivery to bone defect sites [75]. EVs containing these miRNAs promote pre-osteoblast proliferation and migration, stimulate endothelial tube formation, and upregulate osteogenic markers including RUNX2, ALP, and COL1A1. The use of chemically modified oligonucleotides (e.g., locked nucleic acids) further enhances the stability and loading efficiency of nucleic acid cargo in EVs.

Recent innovations include the development of multifunctional EV delivery systems by integrating EVs with nanoparticles, hydrogels, or biodegradable polymers. Hybrid vesicles containing mesoporous silica nanoparticles or polyethylene glycol (PEG) derivatives enable sustained release of osteoinductive signals while preserving EV bioactivity [118]. These systems can be engineered for pH-responsive release, magnetic targeting, or enzyme-mediated activation, providing spatial and temporal control over therapeutic delivery.

Inorganic nanomaterials—including hydroxyapatite, bioglass, and gold nanoparticles—have also been co-delivered with EVs to enhance mineralization and scaffold integration at defect sites [118]. In one study, EVs embedded within hydrogel scaffolds modified with bioactive peptides significantly improved bone repair and neovascularization in a calvarial defect model.

Recent approaches have utilized bacterial EVs (BEVs) engineered to express osteoactive molecules. These BEVs possess intrinsic bone-targeting properties and can be produced on a large scale, offering a novel alternative to mammalian-derived EVs [118]. Engineered BEVs loaded with BMP2 and VEGF modulate the osteogenic–angiogenic axis and reverse osteoporotic bone loss in murine models [118].

Together, these bioengineering strategies highlight the broad potential of post-isolation EV functionalization to generate next-generation osteoinductive therapeutics. These methods offer flexibility, precision, and scalability—qualities essential for clinical translation in bone repair.

### 5.3. Surface Modification for Targeted EV Delivery

Surface modification of EVs enhances their targeting to bone tissue, thereby improving therapeutic specificity. Strategies include conjugating EVs with bone-targeting peptides (e.g., aspartic acid repeats), aptamers, or antibodies against osteoblast-specific markers (e.g., ALP, CD44) [120]. For example, EVs modified with alendronate, a bisphosphonate, display high affinity for hydroxyapatite and exhibit improved retention in bone microenvironments. Lipid raft engineering or PEGylation can increase EV stability and circulation time while reducing clearance by the reticuloendothelial system [121]. While these modifications improve EV homing to fracture sites or osteoporotic lesions, potential immunogenicity and manufacturing complexity require further investigation.

### 5.4. Integration of EVs with 3D Bioprinting and Scaffold Technologies

Integration of EVs with 3D bioprinting and scaffold technologies is transforming bone tissue engineering by enabling precise spatial control and sustained release. Bioprinted scaffolds composed of hydrogels (e.g., gelatin, alginate) or ceramics (e.g., β-tricalcium phosphate) encapsulate EVs to mimic the bone extracellular matrix [122]. For instance, MSC-EVs incorporated into 3D-printed hydroxyapatite scaffolds enhance osteogenesis and angiogenesis in rat calvarial defects via PI3K/Akt signaling. Bioprinting allows customization of scaffold architecture, optimizing porosity and mechanical properties for bone integration. Furthermore, EVs can be co-printed with growth factors or nanoparticles to create multifunctional constructs. Key challenges include preserving EV bioactivity during printing, achieving uniform distribution, and scaling up production for clinical applications [75].

## 6. Challenges and Future Perspectives

### 6.1. Standardization and Large-Scale Production of EVs

EVs are cell-derived vesicles containing nucleic acids, proteins, and lipids. At the microscopic level, they serve as natural carriers for therapeutic agents and drugs. Limitations of traditional nanocarriers—including high immunogenicity and low biocompatibility—can be mitigated by using EVs [123]. EVs also penetrate the blood–brain barrier efficiently and can act as vectors for gene delivery. However, EV research remains highly challenging due to the complex biogenesis of EVs and their considerable heterogeneity in size, composition, and origin [124]. Standardized methods are therefore required to address heterogeneity and minimize pre-analytical and analytical variance in EV research. This heterogeneity results in both intra-batch and inter-batch variations during large-scale EV production.

Standardizing sample collection, storage, and handling procedures is crucial to reducing pre-analytical and pre-isolation variables arising from the complexity and heterogeneity of biological fluids. The following procedures are essential for minimizing artifacts during analysis and downstream isolation.

#### 6.1.1. Sample Collection, Matching, Sample Size, and Data Collection

For isolation of EVs from cultured cells, serum-free medium or EV-free serum should be used as a growth supplement. During clinical blood collection, it is critical to use a needle gauge that minimizes shear forces responsible for platelet activation and subsequent release of platelet- and RBC-derived EVs. The first portion of collected blood should be discarded, as cell debris from venipuncture may contaminate the EV isolate. During specimen collection, harvesting parent cells is recommended to enable molecular matching, profiling, and differential analysis of cellular and EV components. This approach also supports the identification of specific molecular signatures associated with changes in the EV source. The sample size should provide at least 80% statistical power for biomarker identification and EV component profiling.

#### 6.1.2. Sample Handling and Processing

The choice of anticoagulant is critical. Multiple studies recommend EDTA for EV-RNA downstream analysis, as it prevents the formation of EV–cell aggregates and inhibits platelet-derived EV release.

Initial centrifugation separates components such as RBCs, WBCs, and platelets from EVs at speeds that minimize cellular component release. Processing time should be minimized to prevent sample degradation due to elevated temperature and the presence of RNases and proteases.

#### 6.1.3. Sample Stability and Storage

Current evidence indicates that −80 °C is optimal for preserving EV contents for downstream molecular profiling. Freeze–thaw cycles should be minimized, as they may cause EV aggregation and lysis, leading to errors in EV size estimation, underestimation of EV count, and loss of drug during isolation, ultimately reducing potency during drug delivery.

Transitioning EVs from basic research to clinical application requires scalable and reproducible manufacturing processes. Despite rapid advances in EV exploration and evaluation, scaling up EV production for industrial and commercial use remains a substantial challenge [125,126]. Clinical translation of EVs is limited by several factors. First, EV yield is typically less than 1 g of exosomal proteins per 1 mL of culture medium. Second, current isolation methods are time-consuming or costly. Most importantly, no clinically feasible method exists for large-scale EV production with reproducible properties. Consequently, no production protocol currently meets Good Manufacturing Practice (GMP) guidelines.

The EV source is a major determinant in manufacturing. Potential sources include primary mesenchymal stem cells (MSCs), induced pluripotent stem cells, immortalized primary cells (e.g., MSC–MYC), and established cell lines such as HEK293 (human embryonic kidney cells) and CAP (CEVEC Amniocyte Production cells) [127]. Although each source has distinct advantages and limitations, immortalized cell lines are particularly attractive for large-scale EV manufacturing.

##### Upstream Manufacturing Process of EVs

EV production in cell culture (human, microbial, or microalgae) requires optimized processing in bioreactors with precise scaling. Most modern applications utilize primary or immortalized human cells, including MSCs capable of tissue regeneration. Primary cells are typically grown in two-dimensional culture systems with low cell density. Increasingly, 3D bioreactors—such as stirred tanks with microcarriers, packed-bed systems, and hollow-fiber cartridges—are being adopted due to the limited productivity and scalability of 2D systems. Human cell cultivation is compatible with advanced bioprocessing technologies, including fed-batch and high-density perfusion using chemically defined media, which support high EV yields. Additionally, these processes are compatible with existing biomanufacturing infrastructure. Marine microalgae have also been introduced for large-scale EV production, demonstrating strong performance in photobioreactors with ultra-low-cost seawater-based media. Engineering microalgae-derived EVs may further enhance their therapeutic potential [128]. Daily sampling of supernatant is performed to monitor cell growth, viability, and biochemical parameters.

##### Downstream Manufacturing Process of EVs

Downstream techniques can be applied to a broad spectrum of EV types. To enhance EV purity, these processes employ multiple unit operations, typically combined in various sequences. Most methods begin with cell separation using differential centrifugation, filtration, or both. However, centrifugation is slow, labor-intensive, and incompatible with large-scale GMP manufacturing. Therefore, size-exclusion, ion-exchange, and affinity chromatography have been adopted. Newer purification methods—such as field-flow fractionation, nanoscale lateral displacement, and affinity separation—have also been implemented. Size-exclusion chromatography separates small nonvesicular proteins and nucleic acids based on EV properties. Anion and cation exchange chromatography remove larger particles and impurities [127].

### 6.2. Dosing, Stability, and Storage Concerns

The therapeutic efficacy of EVs is routinely evaluated in dosing protocols during both preclinical and clinical trials; however, challenges arise when implementing these protocols post-testing. Dose variations may occur depending on disease model selection, particularly when EV doses are chosen without considering pharmacokinetics or biodistribution. Reliable dosing strategies are essential to ensure accurate measurement of therapeutic efficacy, pharmacokinetics, and dynamics of EV-borne cargo. Nonetheless, EV research still faces inconsistencies in production, isolation methods, quantification, and characterization [129].

To comprehensively evaluate EV dosing strategies, multiple studies are required. These investigations are essential for elucidating EV characterization, identifying disease-specific biomarkers, and assessing the therapeutic efficacy of EVs derived from specific cell sources or engineered EVs. Subsequently, research should address disease-specific applications (e.g., malignancies, cardiovascular, and neurological disorders), dosing frequency, administration route during therapeutic intervention, and the quantity of EVs administered to patients. In vivo EV dosing is typically quantified by total protein content and particle number, which serve as the primary metrics in preclinical studies. Studies examining administration routes indicate that higher doses can be safely delivered systemically (e.g., intravenously or intraperitoneally) compared to local delivery, regardless of the disease model. Current dose conversion factors in therapeutics often rely on simple empirical formulas that do not account for physiological parameters or drug pharmacokinetics. Therefore, these factors must be integrated into EV therapeutic strategies. Dose–response studies are thus critical for refining dose-conversion factors.

Despite significant progress in exploring the therapeutic, drug delivery, and diagnostic potential of EVs, determining optimal EV storage conditions remains challenging. Limited information exists on how storage parameters influence EV integrity both pre- and post-isolation. Storage can affect EV size, molecular content, functionality, and behavior. Storage at –80 °C is widely regarded as optimal for both biofluids and isolated EVs [130], while isolated EVs may be maintained at 4 °C for short-term storage. Lyophilization also plays a key role in preserving EV products. However, substantial challenges remain in understanding the specific effects of storage on EV properties. For instance, one study reported significant EV loss after storage at 4 °C and 20 °C. Additionally, the mean size of exosomes increased following four days of storage at 4 °C. Studies assessing variables such as temperature, freeze–thaw cycles, and pH have yielded inconsistent findings. Recently, phosphate-buffered saline (PBS) has been identified as a suitable medium for EV storage. Table 11 summarizes studies evaluating the impact of different biofluid sources and storage conditions on EV characteristics.

Recent studies have demonstrated that prolonged storage of EVs at 4 °C leads to increased particle size, membrane rupture, aggregation, and protein degradation. Therefore, while 4 °C may be suitable for short-term storage, it is not recommended for long-term preservation, as EV stability declines significantly over time. One study reported no significant difference in EV number when samples were stored at 4 °C or −80 °C for one month [131]. However, several reports indicate that storing EVs at room temperature rapidly reduces particle concentration and integrity within a few days. Storage above 4 °C, including ambient temperature, further compromises EV stability and quality. The duration of storage is also critical for maintaining EV integrity and stability. These effects were observed regardless of temperature, although findings were inconsistent. The polydispersity index and zeta potential are key physical parameters for assessing EV stability and homogeneity during storage [132]. Zeta potential reflects the surface charge of EVs, influencing colloidal stability and aggregation under various storage conditions. Higher zeta potential indicates improved stability and decreased aggregation over time. Changes in zeta potential during storage may signal alterations in EV surface properties or aggregation state [133].

### 6.3. Safety and Immunogenicity of EV-Based Therapies

For further clinical development, it is critical to address the immunogenicity of EVs, beyond their apparent lack of immunotoxicity. Few studies have investigated the immunological recognition of EVs. A comprehensive understanding of EV-induced immunogenicity and clearance is necessary to inform therapeutic strategies, including approaches to minimize immune recognition [134]. Although EV therapeutics and drug delivery platforms have shown favorable safety profiles in preclinical and clinical studies, the potential for unintended immunogenicity remains largely unexplored.

#### 6.3.1. Immune Responses to EVs

The cellular origin of EVs significantly influences their immunogenicity. When EVs are derived from autologous cells, their inherent immunogenicity is generally not a concern. However, EVs can express mismatched histocompatibility complexes, specifically MHC class I and II antigens, which must be considered with allogeneic human sources. These EVs should only be used if they can support or maintain immune regulation. Administration of allogeneic human-derived EVs during pregnancy may trigger pro-inflammatory responses, potentially resulting in serious fetal complications. Thus, EVs can disrupt maternal–fetal communication during pregnancy, and their use should be limited to cases where therapeutic benefits outweigh potential risks. Immunosuppressants are not required for patients receiving cell therapy or processing products expected to provide only transient effects [135]. In contrast, for therapies anticipating long-term engraftment, immunosuppressants are necessary due to potential MHC mismatch. Although EVs are rapidly metabolized, repeated administration of allogeneic EVs necessitates immunosuppression.

#### 6.3.2. Immune Responses to Impurities Present in EVs

EV preparations may contain impurities, including viruses, virus-like particles, microorganisms, mycoplasma, media components and reagents, airborne particles, and artificial microparticles. Quality control measures are essential to minimize immunological reactions to such contaminants. Reducing contamination through careful design and management of manufacturing processes is critical. Accordingly, the use of reagents and recombinant proteins free from animal-derived components, and minimizing raw materials of animal origin, is recommended.

#### 6.3.3. EV-Induced Immune Responses

Immune reactions elicited by EVs may underlie their intended pharmacological effects. For example, immunosuppression is the therapeutic goal of MSC-derived EVs, although this can increase susceptibility to infection. Conversely, DC-derived EVs are designed to stimulate immune responses, which is advantageous for both preventive and therapeutic applications in conditions of immune hyperactivity (e.g., cytokine storm) [136].

DPSC-derived EVs represent a promising alternative for modulating immune responses. Both immune and non-immune cells can release EVs that regulate immunity. The most extensively studied immunological EVs are those derived from tumors and antigen-presenting cells. Tumor-derived EVs inhibit TGF-β-associated macrophage maturation.

### 6.4. Future Directions in EV-Based Bone Regeneration

The prevalence of bone-related disorders continues to rise with the aging population in developing countries, yet current bone regeneration therapies remain suboptimal in many cases. The limited availability of effective treatments has driven researchers to explore innovative strategies, including the use of EVs for bone regeneration [137,138]. Incorporation of EVs with biomaterial carriers can accelerate bone repair and regeneration. The genetic cargo of EVs—including mRNAs and miRNAs—modulates gene expression in target cells, contributing to their regenerative potential.

Blood vessels deliver minerals, growth factors, and progenitor cells to sites of regeneration, supporting tissue homeostasis. The angiogenic capacity of EVs enhances neovascularization, facilitating bone development and repair. Osteoblasts regulate bone formation by producing minerals, primarily calcium and phosphate. EVs directly promote osteoblast activity and proliferation, as observed with prostate cancer cell-derived and bone marrow stromal cell-derived EVs. MSC-derived EVs have demonstrated favorable outcomes in bone regeneration. The anatomical origin and developmental stage of source tissues are key determinants of EV potency.

EV-mediated intercellular communication between osteoblasts and osteoclasts is emerging as a novel regulator of bone remodeling. Osteoblast-derived EVs carrying RANKL (Receptor Activator of Nuclear Factor-κB Ligand) are transferred to osteoclast precursors, promoting osteoclastogenesis via RANKL–RANK signaling. In vitro studies show that osteoblast-derived EVs can enter ST2 recipient cells and induce osteoblastic differentiation through the Wnt signaling pathway, affecting Axin1 and β-catenin levels. EVs modified with tricalcium phosphate further enhance osteogenic differentiation. Additionally, increased osteogenesis in rat calvarial bone defects has been linked to activation of the PI3K/Akt signaling pathway. M2 macrophages are essential for tissue and bone regeneration, and tumor-derived EVs suppress macrophage maturation via TGF-β. Modulation of innate and adaptive immunity by EVs represents a potential therapeutic target in bone regeneration. Notably, miR-196a promotes osteoblastic differentiation, whereas miR-214-3p suppresses osteoblastic bone formation [139].

## 7. Conclusions

Despite notable progress, EV research and therapeutic development face persistent challenges. Heterogeneity in EV composition, driven by differences in parent cell types, isolation techniques, and batch conditions, complicates standardization and reproducibility. The absence of universally accepted nomenclature and protocol standards further hampers the comparison and validation of results [140]. Safety considerations, such as immunogenicity, potential contamination, and off-target effects, underscore the need for robust quality control during EV production and purification. While most studies demonstrate favorable safety profiles, sustained monitoring for adverse events and long-term effects remains critical. Regulatory advances, including adherence to MISEV guidelines and development of reference materials, are essential to improve consistency, transparency, and translation of EV-based interventions [141]. Addressing these limitations will be pivotal for realizing the clinical potential of EVs in bone regeneration and beyond. Although EVs show considerable promise as therapeutic agents for bone degenerative disorders, several key challenges must be addressed before their full clinical potential can be realized. First, elucidating the complex molecular mechanisms and cargo responsible for the osteogenic and bone-modulatory functions of EVs is necessary. This knowledge will guide the development of more effective approaches to enhance their bioactivity and therapeutic efficacy. Second, understanding the mechanisms of EV uptake by target cells and the resulting downstream signaling is critical for functional optimization. Surface modification of EVs is important for targeted drug delivery and protection against degradation. Standardized protocols for EV isolation and characterization are required to produce clinical-grade products [142]. Further research is needed to advance our understanding of EV biology, optimize scalable production and delivery methods, and conduct clinical trials to realize the potential of EVs in bone regeneration.

## Figures and Tables

**Table 1:** Composition and features of exosomes.

Category	Mechanism	Details	Significance	References
Biogenesis markers	ESCRT (endosomal sorting complex required for transport) machinery ESCRT-independent pathway	Includes proteins such as TSG101, Alix, HSC70, and HSP90β Utilizes sphingomyelinase rather than ESCRT	These proteins shape and load exosomes, are present in exosomes from all cell types, and are termed “exosomal markers.” Exosome release persists despite ESCRT knockout, particularly for CD63-positive vesicles.	[29,31,32]
Membrane proteins	Tetraspanins (CD9, CD63, CD81)	Transmembrane proteins highly enriched in exosomes	Previously considered exosome-specific, now also identified in microvesicles and apoptotic bodies	[33,34]
Other surface proteins	Plasma membrane-associated proteins	Widely present in exosomes	Facilitate cargo selection, targeting, and intercellular communication	[35]
Protein content (internal)	Glycoproteins Post-translationally modified proteins	More abundant in exosomes compared to parent cells Includes phosphorylated and extensively glycosylated proteins	Glycosylation enhances cellular interaction and targeting More enriched in microvesicles than in exosomes, aiding vesicle classification	[35,36]
Unexpected proteins	Mitochondrial and nuclear proteins Golgi and ER proteins	Detected due to inter-organelle trafficking Present at low abundance, likely from contact with early endosomes	Challenges prior assumptions that these proteins are absent from exosomes Still classified as non-exosomal markers due to their low abundance relative to the total cell lysate	[29,37]
Cargo sorting proteins	CD63 (also a tetraspanin) Alix and TSG101	Present in both ESCRT-dependent and ESCRT-independent exosomes Directly participate in cargo sorting during vesicle formation	Strong, though not exclusive, exosome marker Commonly used indicators of classical exosome biogenesis	[27,29,32,37]

**Table 2:** List of transcription factors involved in Wnt/β-catenin-mediated osteogenesis.

Transcription Factor	Function	Mechanism
β-catenin	Co-activator	Associates with TCF/LEF in the nucleus to activate osteogenic genes; becomes stabilized following Wnt signaling
2. TCF/LEF (T-cell factor/lymphoid enhancer-binding factor)	DNA-binding proteins	Forms complexes with β-catenin to promote transcription of genes driving osteoblast lineage specification
3. RUNX2 (runt-related transcription factor 2)	Master osteogenic regulator	Activated by Wnt/β-catenin signaling; induces osteoblast differentiation and bone matrix gene expression
4. Osterix (SP7)	Osteoblast-specific factor	Acts downstream of RUNX2; required for the maturation of pre-osteoblasts into functional osteoblasts
5. Dlx5 (distal-less homeobox 5)	Transcriptional regulator	Upregulates RUNX2 expression and facilitates osteogenic differentiation
6. Msx2 (Msh homeobox 2)	Regulator of early osteogenesis	Induced by Wnt signaling; stimulates osteoprogenitor cell proliferation
7. ATF4 (activating transcription factor 4)	Bone matrix regulator	Cooperates with RUNX2 to increase osteocalcin and other matrix protein expression
8. Twist1/2	Negative regulators	Suppress RUNX2; inhibition by Wnt signaling promotes osteogenesis

**Table 3:** List of ligand–receptor pairings.

Ligand	Type II Receptor	Type I Receptor (ALKs)
BMP-2/4/7	BMPR-II, ActR-IIA, ActR-IIB	ALK3 (BMPR-IA), ALK6 (BMPR-IB)
TGF-β1/β2/β3	TGF-βRII	ALK5 (TGF-βRI), ALK1 (endothelial cells)

**Table 4:** Key upregulated osteogenic genes and their functions.

Gene	Role in Osteogenesis
Runx2	Drives osteoblast lineage commitment
Osterix	Essential for osteoblast maturation
ALP	Promotes matrix mineralization
Osteocalcin	Mediates calcium binding in bone
Type I Collagen	Primary structural protein in bone matrix

**Table 5:** Three possible fates of osteoblasts.

Fate	Description
Osteocytes	Embedded in mineralized matrix; maintain bone homeostasis
Bone lining cells	Quiescent, flattened osteoblasts on bone surfaces
Apoptosis	Undergo programmed cell death when further activity is unnecessary

**Table 6:** Negative regulators and their mechanisms.

Mechanism	Regulators	Key Effects
Extracellular inhibitors	Noggin, Chordin, Gremlin	Prevent ligand–receptor interaction
Inhibitory SMADs	SMAD6, SMAD7	Inhibit R-SMADs; promote receptor degradation
Ubiquitin ligases	Smurf1, Smurf2, NEDD4L	Target receptors or SMADs for degradation
Receptor endocytosis	Caveolin-mediated pathways	Internalize receptors from cell surface
MicroRNAs	miR-26a, miR-145, miR-20a	Suppress pathway gene expression post-transcriptionally
Feedback loops	SMAD6/7, Noggin (signaling-induced)	Self-regulate signaling intensity

**Table 7:** Summary of Akt activation downstream effects and key transcription factors [81].

Biological Process	Transcription Factor/Target	Akt-Mediated Effect
Apoptosis inhibition	BAD	Phosphorylation at Ser136 promotes 14-3-3 binding, sequestering BAD in the cytoplasm and preventing apoptosis induction
	Caspase	Phosphorylation suppresses protease activity, thereby blocking apoptosis initiation
	FOXO1/3a/4	Phosphorylation retains FOXO in the cytoplasm, suppressing pro-apoptotic gene transcription
	NF-Κb	Activation induces transcription of genes promoting survival and proliferation
B. Cell proliferation	mTORC1	Activation stimulates protein synthesis and cellular growth
	GSK-3β	Inhibition stabilizes cyclin D1, advancing cell cycle progression
	E2F1	Akt-dependent phosphorylation modulates E2F1, affecting cell cycle advancement
C. Metabolic regulation	FOXO1	Inhibition decreases gluconeogenic gene expression, reducing glucose output
	SREBP-1c	Activation upregulates genes involved in lipid biosynthesis
D. Angiogenesis	HIF-1α	Stabilization and activation induce angiogenic gene expression under hypoxic conditions
E. Autophagy regulation	TEFB	Phosphorylation causes cytoplasmic retention, diminishing lysosomal biogenesis and autophagy
F. Cell cycle progression	p21	Phosphorylation at Thr145 restricts cell cycle progression
G. Transcription regulation	CREB	Phosphorylation promotes transcription of genes involved in survival and metabolism
	STAT3	Phosphorylation increases expression of genes governing cell growth and survival
	Runx2	Phosphorylation enhances invasive gene expression associated with cell growth and survival
	YAP	Phosphorylation at Ser127 results in cytoplasmic retention and reduced transcriptional activity

**Table 8:** Core MAPK module: sequential phosphorylation cascade.

Kinase Tier	Components	Functions	Ref.
MAPKKK	RAF (A-RAF, B-RAF, C-RAF)	Activated by RAS-GTP; phosphorylates and activates MAPKK (MEK)	[83]
MAPKK	MEK1/2	Dual-specificity kinase; phosphorylates ERK1/2 on Thr and Tyr residues	[84]
MAPK	ERK1/2	Activated ERK translocates to the nucleus and regulates transcription	[83]

**Table 9:** Key transcription factors and their mechanisms [85].

Transcription Factors	Target Genes	Mechanism
c-FOS/c-JUN (AP-1)	Cyclin D1, Bcl-2	AP-1 facilitates cell cycle progression and upregulates anti-apoptotic genes.
2. Elk-1	c-Fos	ERK-mediated phosphorylation activates Elk-1, which binds the serum response element (SRE) to induce c-Fos transcription.
3. CREB	Survivin, Mcl-1	Phosphorylated CREB promotes cell survival by inducing anti-apoptotic gene expression.
4. MYC	E2F, Cyclin E	MYC promotes G1/S transition by activating cyclins and repressing cyclin-dependent kinase inhibitors.

**Table 10:** Key EV types in osteogenesis. Below is a summary table comparing major EV types relevant to bone regeneration, their cellular origins, associated bioactive cargo, and osteogenic effects [114].

EV Type	Parent Cell Origin	Bioactive Cargo	Osteogenic Effects
Exosomes	MSCs, osteoblasts	Proteins (RUNX2, BMPs), miRNAs (miR-21, miR-26a, miR-29b), lipids	Promote osteoblast differentiation; stimulate bone matrix synthesis; enhance angiogenesis; modulate immune cell phenotype
Microvesicles	Osteoblasts, ECs	Protein kinases, mRNAs, miRNAs (miR-210)	Support mineralization; enhance osteogenic gene expression; facilitate matrix remodeling
EC-derived EVs (EC-EVs)	Endothelial cells	VEGF, FGF2, Ang1, miR-126, MMPs	Couple angiogenesis and osteogenesis; promote vascularization; stimulate MSC differentiation
Macrophage-derived EVs (Mφ-EVs)	M2 macrophages	Anti-inflammatory cytokines, miRNAs	Modulate inflammation; induce osteogenesis and bone repair in inflammatory/aged tissue
Stress EVs	Bone/progenitor cells	Stress-adaptive proteins, regulatory RNAs	Adaptation to pathological stress: potential modulation of repair processes
Matrix vesicles	Osteoblasts	Mineralization proteins, enzymes (ALP)	Initiate and regulate mineral deposition in bone
Oncosomes	Cancer cells	Tumor-promoting proteins, oncogenic miRNAs	Not osteogenic; may disrupt bone homeostasis

**Table 11:** Summary of EV sources, types, storage conditions, and observed changes.

Sl. No.	EV Source and Type	Storage Temperature	Storage Duration	Particle Changes Observed	Content Changes Observed
1.	Blood (sEVs)	RT, 4 °C, 20 °C, 40 °C, 80 °C, 160 °C	Days or months	NA	Long-term storage at RT and 4 °C increased signal intensity, while short-term storage reduced signal intensity
2.	Plasma (EVs)	–80 °C; single freeze–thaw cycle	12–20 m	EV levels decreased over time; single freeze–thaw cycle increased EV count	NA
3.	Platelet (nanovesicles)	–80 °C + DMSO	1 h	Nanovesicle number increased	NA
4.	Milk (EVs)	4 °C to 80 °C	2–8 weeks	NA	CD63 and CD9 expression remained unchanged
5.	Urine (exosomes)	RT, 4 °C to 80 °C	2 h, 1 day, 1 week	EV yield declined over time	NA
6.	Plasma	4 °C, 20 °C to 80 °C	2 weeks to 2 years	NA	RNA levels decreased after weeks at 4 °C; freezing did not affect RNA or protein levels
7.	Serum	RT, 4 °C	6–168 h	NA	RT for 24 h and 4 °C for 1 week did not alter CD63, TSG101, or DNA concentration
		Freeze–thaw cycles	1, 3, and 5 cycles	NA	CD63 and TSG101 unchanged; DNA concentration markedly decreased

Note: NA-It means no studies were found for the related EV source and type.

## Data Availability

No data were used for the research described in this article.
