# Correction: Biswas et al. Extracellular Vesicles in Osteogenesis: A Comprehensive Review of Mechanisms and Therapeutic Potential for Bone Regeneration. *Curr. Issues Mol. Biol.* 2025, *47*, 675

**DOI:** 10.3390/cimb48020154

**Published:** 2026-01-30

**Authors:** Sreyee Biswas, Prakash Gangadaran, Chandrajeet Dhara, Shreya Ghosh, Soumya Deep Phadikar, Akash Chakraborty, Atharva Anand Mahajan, Ranit Mondal, Debdeep Chattopadhyay, Trisha Banerjee, Anuvab Dey, Subhrojyoti Ghosh, Anand Krishnan, Byeong-Cheol Ahn, Ramya Lakshmi Rajendran

**Affiliations:** 1 Department of Biotechnology, Heritage Institute of Technology, Kolkata 700107, West Bengal, India; sreyeebiswas@gmail.com; 2 Department of Nuclear Medicine, School of Medicine, Kyungpook National University, Daegu 41944, Republic of Korea; prakashg@knu.ac.kr; 3 Cardiovascular Research Institute, Kyungpook National University, Daegu 41944, Republic of Korea; 4 School of Biosciences, Apeejay Stya University, Sohna-Palwal Road, Sohna, Gurugram 122103, Haryana, India; chandudhara2804@gmail.com; 5 Department of Microbiology, St. Xavier’s College, Kolkata 700016, West Bengal, India; shreyaghosh11813@gmail.com (S.G.); trishabanerjee947@gmail.com (T.B.); 6 Department of Chemistry and Chemical Biology, Indian Institute of Technology (ISM), Dhanbad 826004, Jharkhand, India; soumyadeepphadikar3@gmail.com; 7 Department of Biotechnology, Indian Institute of Technology Madras, Chennai 600036, Tamil Nadu, India; akashchakraborty138@gmail.com (A.C.); subhrojyotighosh8@gmail.com (S.G.); 8 Advanced Centre for Treatment, Research and Education in Cancer, Navi Mumbai 410210, Maharashtra, India; atharva66mahajan@gmail.com; 9 Department of Pharmaceutical Technology, Jadavpur University, Kolkata 700032, West Bengal, India; ranitmondal30@gmail.com; 10 Department of Biotechnology, St. Xavier’s College, Kolkata 700016, West Bengal, India; chattopadhyay.debdeep777@gmail.com; 11 Department of Biosciences and Bioengineering, Indian Institute of Technology Guwahati, North Guwahati 781039, Assam, India; anuvab2000dey@gmail.com; 12 Precision Medicine and Integrated Nano-Diagnostics (P-MIND) Research Group, Office of the Dean, Faculty of Health Sciences, University of the Free State, Bloemfontein 9300, South Africa; krishnana1@ufs.ac.za; 13 BK21 FOUR KNU Convergence Educational Program of Biomedical Sciences for Creative Future Talents, Department of Biomedical Sciences, School of Medicine, Kyungpook National University, Daegu 41944, Republic of Korea; 14 Department of Nuclear Medicine, Kyungpook National University Hospital, Daegu 41944, Republic of Korea

## 1. Figure Legend

In the original publication [1], there was a mistake in the legend for Figures 1–3. The BioRender has not been properly cited for the figures created using BioRender. The correct legend appears below.

**Figure 1. Mechanisms of EV–cell interaction.** Mechanisms of extracellular vesicle (EV)–cell interaction. EVs interact with target cells via Receptor-Mediated Interactions, involving ligand binding and Membrane Fusion Pathway. EVs: extracellular vesicles; HSP70: heat shock protein 70; TLR4: toll-like receptor 4; p38: p38 mitogen-activated protein kinase; ERK: extracellular signal-regulated kinase; FAK: focal adhesion kinase; MAPK: mitogen-activated protein kinase; MMP-9: matrix metalloproteinase-9; DKK1: Dickkopf-related protein 1; SNAREs: soluble N-ethylmaleimide-sensitive factor attachment protein receptors; R18: octadecyl rhodamine B chloride (fluorescent probe); miRNA: microRNA. *Created in BioRender. Gangadaran, P. (2025) BioRender.com/9uyghiq*.

**Figure 2. Illustrations of different types of extracellular vesicles in the bone microenvironment.** EVs: extracellular vesicles; MSC-EVs: mesenchymal stem cell-derived extracellular vesicles; OB-EVs: osteoblast-derived extracellular vesicles; EC-EVs: endothelial cell-derived extracellular vesicles; M_2_ Macrophage-EVs: type 2 macrophage extracellular vesicles; Platelet-EVs: platelet-derived extracellular vesicles; miRNAs: microRNAs; mRNAs: messenger RNAs. *Created in BioRender. Gangadaran, P. (2025) BioRender.com/g8k2s3b*.

**Figure 3. Schematic representation of the canonical Wnt/β-catenin signaling pathway in osteogenesis.** The binding of Wnt ligands to their receptors inhibits β-catenin degradation, allowing its accumulation and translocation into the nucleus. Nuclear β-catenin activates target gene transcription, which promotes osteoblast differentiation and subsequent bone matrix production. *Created in BioRender. Gangadaran, P. (2025) BioRender.com/y5madsl*.

## 2. Error in Figure

In the original publication [1], there was a mistake in Figure 2 as published. A draft version of Figure 2 (AI-generated) was submitted in error. The corrected Figure 2 appears below.

The authors state that the scientific conclusions are unaffected. This correction was approved by the Academic Editor. The original publication has also been updated.

## Figures and Tables

**Figure 2:**
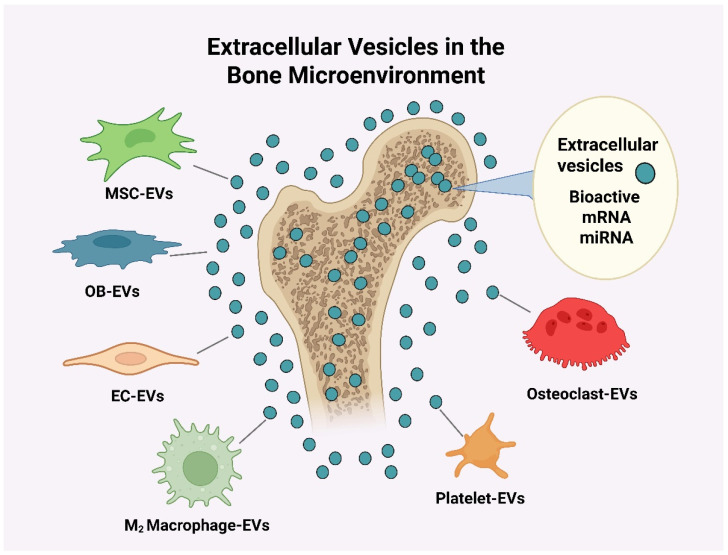
**Illustrations of different types of extracellular vesicles in the bone microenvironment.** EVs: extracellular vesicles; MSC-EVs: mesenchymal stem cell-derived extracellular vesicles; OB-EVs: osteoblast-derived extracellular vesicles; EC-EVs: endothelial cell-derived extracellular vesicles; M_2_ Macrophage-EVs: type 2 macrophage extracellular vesicles; Platelet-EVs: platelet-derived extracellular vesicles; miRNAs: microRNAs; mRNAs: messenger RNAs. *Created in BioRender. Gangadaran, P. (2025) BioRender.com/g8k2s3b*.
